# Infectious Complications and Toxicities of the Teclistamab Plus Daratumumab Combination in Relapsed or Refractory Multiple Myeloma: A Disproportionality and Drug–Interaction Analysis of the FDA Adverse Event Reporting System

**DOI:** 10.3390/cancers18203388

**Published:** 2026-10-20

**Authors:** Carmel Awadallah, Sohaib Irfan, Al-Ola Abdallah, Muhammad Bilal Abid

**Affiliations:** 1 Department of Internal Medicine, St. John’s Episcopal Hospital, New York, NY 11691, USA; 2 Department of Internal Medicine, Landmark Medical Center, Woonsocket, RI 02895, USA; 3 Division of Hematologic Malignancies and Cellular Therapeutics, Department of Internal Medicine, University of Kansas Medical Center, Kansas City, KS 66160, USA; 4 Stem Cell Transplantation and Cellular Immunotherapy Program, Department of Hematology/Oncology, UMC TLC2 Foundation Cancer Center, Texas Tech University Health Sciences Center, Lubbock, TX 79430, USA

**Keywords:** multiple myeloma, teclistamab, daratumumab, bispecific antibodies, B-cell maturation antigen, pharmacovigilance, FAERS, drug–drug interaction, infections, immunotherapy toxicity, supportive care

## Abstract

**Background:** Teclistamab plus daratumumab (Tec-Dara) was approved in March 2026 for relapsed or refractory multiple myeloma, but infections are frequent. We hypothesized that dual BCMA- and CD38-directed immune depletion causes infectious toxicity exceeding that expected from either agent alone. **Methods:** In the FDA Adverse Event Reporting System (FAERS; Q4 2022–Q4 2025; all combination reports pre-approval), we compared reports listing both agents (*n* = 353) with teclistamab-only (*n* = 2774) and daratumumab-only (*n* = 19,230) reports. Interaction was quantified as Ω = ROR_combination_/(ROR_teclistamab_ × ROR_daratumumab_), where ROR is the reporting odds ratio, with sensitivity analyses addressing calendar period, sparse counts, comparator, and confounding. **Results:** Pneumonia (Ω, 1.50; 95% CI, 1.06–2.12) and influenza (Ω, 2.63; 95% CI, 1.20–5.77) were reported more often than expected. The pneumonia estimate increased against a myeloma-restricted background and after covariate adjustment, but was no longer statistically significant after excluding reports co-listing immunomodulatory drugs or proteasome inhibitors or with a relapsed-disease daratumumab comparator. Cytokine release syndrome, neurotoxicity, and cytopenias were reported less often than expected, a reporting pattern rather than protection. A bone marrow failure signal rested on few reports and is hypothesis-generating only. **Conclusions:** Consistent with our hypothesis, Tec-Dara was associated with higher-than-expected reporting of respiratory infections, although confounding by treatment population cannot be excluded. Any new respiratory symptom or fever warrants same-day evaluation.

## Simple Summary

Teclistamab and daratumumab are two immunotherapies for multiple myeloma that were approved as a combination in March 2026. The combination markedly prolongs disease control but causes frequent and sometimes fatal infections. Using more than 4.6 million safety reports submitted to the U.S. Food and Drug Administration between late 2022 and 2025, all before the combination was approved, we asked whether side effects are reported with the combination more often than expected from either drug alone. Pneumonia and influenza were reported more often than expected, although the pneumonia finding weakened in some analyses that accounted for differences between patient groups. Cytokine release syndrome, neurotoxicity, and low blood counts were reported less often than expected; this reflects how these well-known side effects are reported and does not mean that the combination lowers their risk. Any new respiratory symptom or fever in a patient receiving this combination should be evaluated the same day.

## 1. Introduction

Anti-CD38 monoclonal antibodies and B-cell maturation antigen (BCMA)-directed bispecific T-cell engagers have changed the treatment of multiple myeloma (MM) [1]. Daratumumab, a human IgGκ anti-CD38 antibody, is used across multiple lines of therapy, whereas teclistamab, the first approved BCMA × CD3 bispecific antibody, produces deep and durable responses in heavily pretreated disease [1]. Because these agents act through complementary yet distinct immune mechanisms involving CD38-mediated plasma cell depletion and BCMA-directed T-cell redirection, there is a strong rationale for combining them.

This rationale was confirmed by the phase III MajesTEC-3 trial, in which the teclistamab-plus-daratumumab combination (Tec-Dara) achieved a 36-month progression-free survival of 83.4% versus 29.7% with standard of care (SoC) (hazard ratio, 0.17) and a 36-month overall survival of 83.3% versus 65.0% against daratumumab-based SoC regimens, leading to U.S. Food and Drug Administration (FDA) approval of the Tec-Dara regimen in March 2026 [2,3].

Despite this efficacy, treatment was associated with substantial infectious toxicity, requiring protocol modification and the incorporation of intravenous immunoglobulin (IVIG) replacement for infection prevention. In MajesTEC-3, infections occurred in 96.5% of combination-treated patients (54.1% grade ≥ 3), serious adverse events in 70.7%, and treatment-attributed death in 7.1% of patients [2]. These observations are consistent with the broader literature showing that BCMA-directed bispecific antibodies carry a disproportionate infectious burden, driven by profound hypogammaglobulinemia, T-cell exhaustion, and neutropenia, with pooled all-grade infection rates of approximately 56% and grade ≥ 3 rates near 24% [4,5]. Although increased infectious toxicity with Tec-Dara has been recognized, it remains unknown whether these adverse events reflect the additive effects of two immunosuppressive therapies or reflect an interaction between dual BCMA- and CD38-directed immune targeting. Identifying toxicities that exceed those expected from either agent has important clinical implications because these events may require preventive strategies beyond those recommended for either drug. Because CD38-directed therapy independently depletes immune effector populations, dual immune targeting may amplify infectious complications beyond those expected from either agent alone, and the immunological consequences of combining the two classes cannot be predicted from single-agent experience.

As BCMA-directed combinations move into earlier lines of therapy, where patients may remain on treatment for years, characterizing their toxicity profile has become increasingly important, and real-world safety data for the newly approved combinations are urgently needed. Although randomized clinical trials are well-suited to evaluate efficacy and common adverse events, they are generally underpowered to detect uncommon interaction toxicities or determine whether adverse events exceed what would be expected from the individual agents. For the treating hematologist–oncologist, the practical question is not only how often infections occur with Tec-Dara, but which complications warrant regimen-specific supportive care beyond that already recommended for BCMA-directed bispecific antibodies, including immunoglobulin replacement, antimicrobial and antiviral prophylaxis, vaccination, and dosing-interval decisions. This question is increasingly pressing because the regimen is approved for patients who have received at least one prior line of therapy, including a proteasome inhibitor and an immunomodulatory agent [3], and other bispecific antibody–anti-CD38 combinations are in phase III development.

Large pharmacovigilance databases provide an opportunity to evaluate real-world safety signals that are difficult to identify in clinical trials. Although spontaneous reporting systems cannot establish incidence or causality, they represent a powerful hypothesis-generating approach for detecting unexpected safety signals after regulatory approval. Previous pharmacovigilance studies have evaluated the safety profiles of individual BCMA-directed bispecific antibodies; however, no study has quantitatively evaluated the interaction toxicity associated with teclistamab and daratumumab combination therapy.

Accordingly, we performed a pharmacovigilance analysis of the FDA Adverse Event Reporting System (FAERS) using a drug–drug interaction framework to determine whether adverse events, particularly infectious complications, are reported with the Tec-Dara combination more often than expected from reports of teclistamab and daratumumab individually, and to identify toxicity domains that should be prioritized in supportive-care protocols and in safety monitoring of ongoing myeloma combination trials. We hypothesized that dual BCMA- and CD38-directed immune depletion causes infectious toxicity exceeding that expected from either agent alone, which would appear as higher-than-expected reporting of infections with the combination. The analysis addresses the toxicity component of the benefit–risk balance only; it is not designed to quantify net clinical benefit.

## 2. Materials and Methods

### 2.1. Data Source, Study Period, and Deduplication

We analyzed the publicly available FAERS quarterly data extract files (DEMO, DRUG, REAC, OUTC, RPSR, and INDI) from the fourth quarter of 2022 (Q4 2022) to the fourth quarter of 2025 (Q4 2025). The start of the window corresponds to the FDA accelerated approval of teclistamab (25 October 2022), the first quarter in which both agents were commercially available in the United States; the end corresponds to the last complete calendar quarter before FDA approval of the combination (5 March 2026), so that the analysis covers the pre-approval period in full.

Because the Tec-Dara regimen was approved by the FDA only in March 2026, after the end of the study window, all combination reports analyzed here describe exposure before approval of the combination. Such reports can originate from interventional studies (e.g., MajesTEC-3 [2] and other teclistamab–daratumumab-containing trials), expanded-access programs, or off-label concurrent use of the two individually marketed agents. The analysis therefore characterizes the pre-approval reporting profile of the combination rather than postmarketing experience with the approved regimen. The public FAERS extracts do not identify clinical-study reports; the reporting characteristics of each cohort (report type, literature-based reports, reporter occupation, and country) are therefore described in Section 3.1.

#### Deduplication Followed FDA Recommendations

For each CASEID, only the most recent version (latest FDA_DT, with ties broken by the highest case version) was retained across all 13 quarterly files, and each retained report was linked to the drug, reaction, outcome, and indication records of that version. This yielded 4,632,365 unique cases across all drugs.

### 2.2. Drug-Name Harmonization and Exposure Cohorts

#### 2.2.1. Drug-Name Harmonization

Drug entries were identified by searching both the verbatim product name (DRUGNAME) and the active-ingredient field (PROD_AI) using case-insensitive text matching. Teclistamab was captured by “teclistamab”, “Tecvayli”, and “teclistamab-cqyv”, and daratumumab by “daratumumab”, “Darzalex” (which also captures Darzalex Faspro), and “hyaluronidase-fihj”, with intravenous and subcutaneous formulations pooled. Within each report, multiple entries for the same agent (e.g., separate doses, cycles, or both formulations) were collapsed into a single exposure indicator, and duplicate Preferred Terms were collapsed, so that each report contributed at most once to each drug and each Preferred Term.

#### 2.2.2. Exposure Cohorts and Drug Roles

FAERS assigns each drug a role code: primary suspect (PS), secondary suspect (SS), concomitant (C), or interacting (I). In the primary analysis, a drug was considered reported if it appeared in any role, because in multi-agent regimens, the partner drug is frequently coded as concomitant even when it is administered as part of the same regimen. Four mutually exclusive, report-level cohorts were defined: (i) combination (Tec-Dara) reports listing both teclistamab and daratumumab (*n* = 353); (ii) teclistamab-only reports listing teclistamab but not daratumumab (*n* = 2774); (iii) daratumumab-only reports listing daratumumab but not teclistamab (*n* = 19,230); and (iv) background, consisting of all remaining reports listing neither agent (*n* = 4,610,008), irrespective of indication or therapeutic class. “Combination” therefore denotes the co-reporting of both agents within a single case report; because administration dates are incomplete in FAERS, concurrent administration could not be verified at the patient level. Similarly, “teclistamab-only” and “daratumumab-only” denote the absence of the other agent of interest and do not exclude other co-reported anti-myeloma drugs (e.g., lenalidomide, pomalidomide, bortezomib, or dexamethasone); their co-reporting in the combination cohort is described in Section 3.1. Drug-role restrictions were examined in a sensitivity analysis (Section 2.5).

Adverse events were coded according to the Medical Dictionary for Regulatory Activities (MedDRA) at the Preferred Term (PT) level (version 29.0). For descriptive purposes, Table 1, aggregate infection reports were defined as reports containing at least one PT within the System Organ Class “Infections and infestations”, and aggregate cytopenia reports as reports containing at least one of the PTs neutropenia, febrile neutropenia, thrombocytopenia, anemia, leukopenia, or pancytopenia (Supplementary Methods). All disproportionality and interaction analyses were performed at the level of individual PTs.

### 2.3. Disproportionality Analysis

For each exposure cohort and each MedDRA PT, reports were cross-classified in a 2 × 2 contingency table against the background cohort (Table 2). The reporting odds ratio (ROR) and proportional reporting ratio (PRR) were calculated as ROR = (a/b)/(c/d) = ad/bc and PRR = [a/(a + b)]/[c/(c + d)]. The 95% confidence interval (CI) of the ROR was obtained with the logit (Woolf) method: SE(ln ROR) = √(1/a + 1/b + 1/c + 1/d), 95% CI = exp(ln ROR ± 1.96 × SE). Estimates were computed for PTs reported in at least three combination reports, a threshold commonly used in pharmacovigilance signal detection to improve estimate stability; a PT was considered disproportionately reported when a ≥ 3 and the lower 95% confidence limit of the ROR exceeded 1. Because every analyzed cell contained at least three reports in the primary analysis, no continuity correction was required; a continuity-corrected analysis is described in Section 2.5. PRRs are reported in Table S2. The RORs and PRRs are complementary measures of disproportionate reporting. ROR was selected as the primary metric because of its statistical advantages and compatibility with interaction modeling [6,7]. Statistical analyses were performed in Python (version 3.11) using pandas (version 3.0), NumPy (version 2.4), and statsmodels (version 0.15).

### 2.4. Drug–Drug Interaction (Ω) Analysis

Among the methods for detecting drug–drug interactions in spontaneous reporting data reviewed by Noguchi et al. [8], we used the multiplicative interaction model described by Thakrar et al. [9]: Ω = ROR_combo_/(ROR_tec_ × ROR_dara_), where ROR_combo_, ROR_tec_, and ROR_dara_ are the RORs of the combination, teclistamab-only, and daratumumab-only cohorts, each computed against the same background (Table 2). Ω is algebraically identical to the exponentiated interaction coefficient of a saturated logistic regression model containing indicators for teclistamab, daratumumab, and their product; Ω = 1 corresponds to the reporting odds expected if the two agents’ associations with the event combined multiplicatively. Because ln Ω = logit(p_combo_) − logit(p_tec_) − logit(p_dara_) + logit(p_background_) and the four cohorts are disjoint, its variance was obtained as Var(ln Ω) = 1/a_combo_ + 1/b_combo_ + 1/a_tec_ + 1/b_tec_ + 1/a_dara_ + 1/b_dara_ + 1/c + 1/d, and the 95% CI as exp(ln Ω ± 1.96 × √Var). Ω was computed only for PTs with at least three reports in each of the three exposure cohorts. A lower 95% confidence limit above 1 was classified as higher-than-expected reporting, an upper limit below 1 as lower-than-expected reporting, and an interval including 1 as no departure from the multiplicative expectation. We use these descriptive terms rather than “synergy” and “antagonism” because Ω describes the relative frequency with which events are reported, not the underlying risk; in particular, Ω < 1 does not imply that either agent mitigates the toxicity of the other [10].

This ratio-based statistic should be distinguished from the shrinkage Ω measure of Norén et al. [11], which evaluates departure from an additive model of reporting risk and is designed for sparse data; the latter was applied as a sensitivity analysis (Section 2.5). A worked example of all calculations is provided in the Supplementary Methods, and the analytic code is available from the corresponding author on reasonable request.

### 2.5. Sensitivity Analyses

#### 2.5.1. Contemporaneous Window (Prespecified)

**Figure 1:**
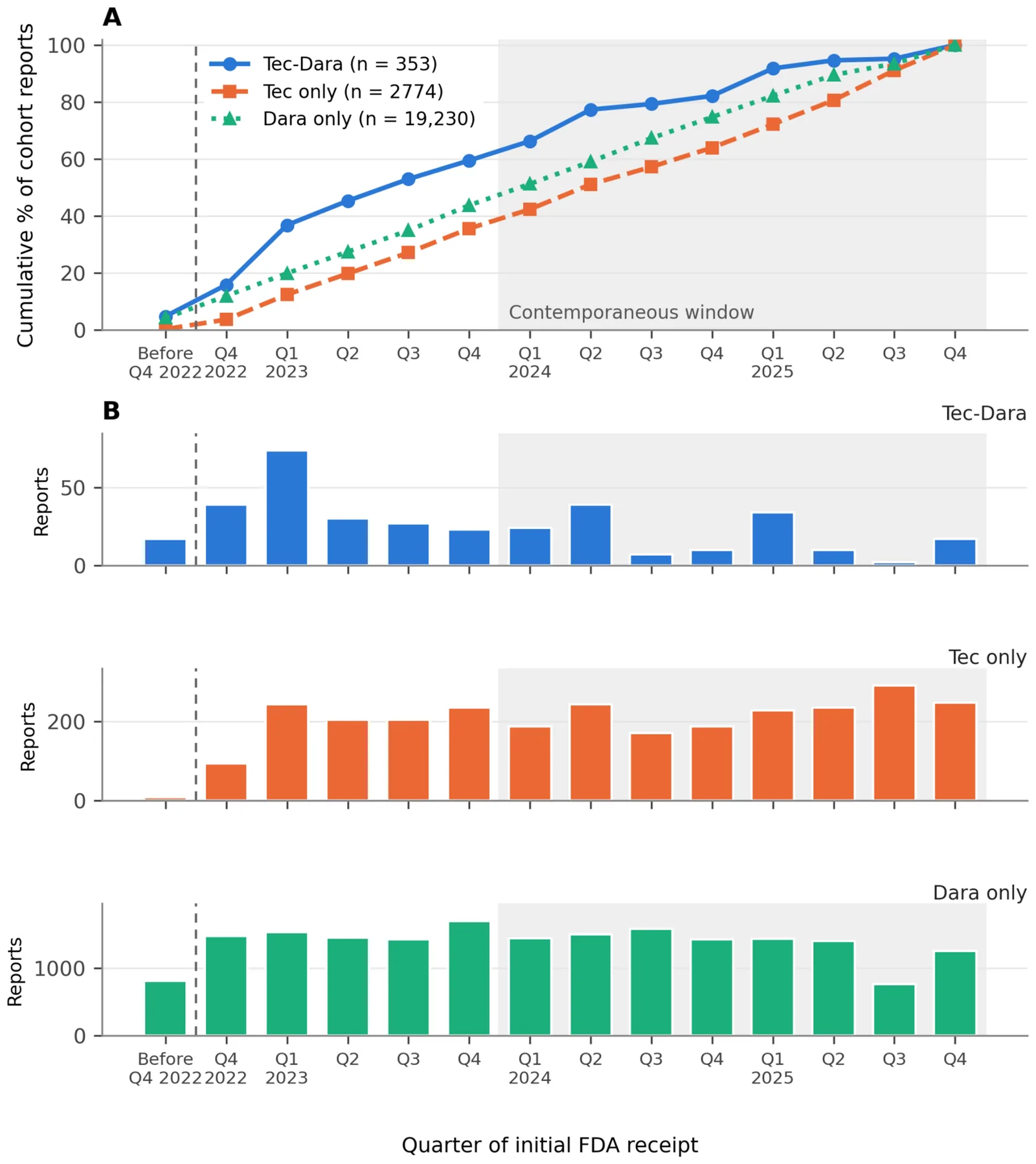
Quarterly accumulation of FAERS reports by exposure cohort, Q4 2022–Q4 2025. (**A**) Cumulative percentage of each cohort’s reports received by quarter, shown on a common scale so that the timing of reporting can be compared across cohorts. (**B**) Number of reports received per quarter in each cohort, shown in separate panels with their own y-axes. Quarters are assigned by initial FDA receipt date; reports first received before Q4 2022 whose latest version fell within the study window are shown as a single initial category. The shaded area marks the contemporaneous window used in the sensitivity analysis (Q1 2024–Q4 2025), and the dashed line marks the start of the study window, the quarter of teclistamab approval (October 2022). The combination was approved in March 2026, after the study window.

Teclistamab entered the market at the start of the study window, whereas daratumumab had been marketed since 2015, and combination reports accrued mainly in 2023, with only 40.5% received in 2024–2025 (Figure 1). The three cohorts therefore differ in the calendar distribution of their reports, and early post-launch reporting (the Weber effect), secular changes in reporting (e.g., of COVID-19-related terms), and publicity surrounding MajesTEC-3 could bias their comparison. We therefore restricted all cohorts, including the background, to a contemporaneous window (Q1 2024–Q4 2025). To allow the Weber effect and other reporting trends to be assessed directly, the number of reports received per quarter in each cohort, by initial FDA receipt date, is shown in Figure 1.

#### 2.5.2. Sparse Counts

Because several interaction estimates rest on few reports, three complementary checks were performed: (i) the shrinkage Ω of Norén et al. [11], Ω_N_ = log_2_[(n_111_ + 0.5)/(E_111_ + 0.5)], where n_111_ is the observed number of combination reports with the event and E_111_ = n_11_ × g_11_ is the number expected under an additive model of the event odds, with g_11_ = 1 − 1/[max(o_00_, o_10_) + max(o_00_, o_01_) − o_00_ + 1] and o_00_, o_10_, and o_01_ the event odds in the background, teclistamab-only, and daratumumab-only cohorts; its lower 95% credibility limit was approximated as Ω_025_ = Ω_N_ − 1.96/(ln 2 × √n_111_), Ω_025_ > 0 was classified as higher-than-expected, and Ω_975_ < 0 (Ω_975_ = Ω_N_ + 1.96/(ln 2 × √n_111_)) as lower-than-expected reporting; (ii) recalculation of all RORs and Ω with the Haldane–Anscombe correction (0.5 added to every cell); and (iii) restriction to PTs with at least five reports in each exposure cohort. A classification was considered robust to sparse counts when it was unchanged across these approaches.

#### 2.5.3. Drug Role

The analysis was repeated after restricting exposure to suspect roles: the combination cohort to reports in which both agents were coded PS or SS, and the single-agent cohorts to reports in which the agent of interest was coded PS or SS.

#### 2.5.4. Clinically Aligned Comparator

The full-database background consists predominantly of reports from patients without cancer, which can inflate RORs for events common in hematologic malignancy. We therefore repeated the analysis with the background restricted to reports from patients with multiple myeloma, defined as reports with an indication PT of “Plasma cell myeloma” (or related PTs containing “plasma cell myeloma” or “multiple myeloma”) or listing at least one other anti-myeloma agent (lenalidomide, pomalidomide, thalidomide, bortezomib, carfilzomib, ixazomib, isatuximab, elotuzumab, elranatamab, talquetamab, linvoseltamab, idecabtagene vicleucel, ciltacabtagene autoleucel, belantamab mafodotin, selinexor, or melphalan flufenamide), and listing neither teclistamab nor daratumumab. In a further analysis, reports co-listing an immunomodulatory drug (lenalidomide, pomalidomide, or thalidomide) or a proteasome inhibitor (bortezomib, carfilzomib, or ixazomib) were excluded from all exposure cohorts to reduce confounding by partner agents.

#### 2.5.5. Report Source

The public FAERS extracts do not identify clinical-study reports (the report-source file contains no study code for these cohorts), so the interaction analysis could not be stratified by reporting channel; report type and literature-based reports are described by cohort in Table 1.

#### 2.5.6. Treatment Population

Because infection risk depends on patient characteristics that differ between cohorts, two further analyses addressed residual confounding by treatment population. First, within the myeloma-restricted population, a multivariable logistic regression model for each primary PT included indicators for teclistamab, daratumumab, and their product. It was adjusted for age group, sex, year of initial receipt, report type (expedited, periodic, direct, or literature-based), reporter region, and co-reported anti-myeloma drug classes (immunomodulatory drugs, proteasome inhibitors, and corticosteroids), the last serving as a partial proxy for regimen and line of therapy; the exponentiated interaction coefficient provided a covariate-adjusted Ω. Reports with missing covariates were assigned to a missing-indicator category. Second, to align the daratumumab comparator with relapsed disease, the daratumumab-only cohort was restricted to reports co-listing pomalidomide or carfilzomib, agents used in relapsed disease and including a daratumumab-based comparator regimen of MajesTEC-3 [2].

#### 2.5.7. Positive and Negative Controls

To assess the validity of the method, labeled adverse reactions of each agent served as positive controls for the disproportionality analysis: cytokine release syndrome (CRS), immune effector cell-associated neurotoxicity syndrome (ICANS), and hypogammaglobulinemia for teclistamab, and neutropenia and thrombocytopenia for daratumumab. Four PTs not expected to be associated with either agent or with their combination (musculoskeletal pain, headache, nausea, and fatigue) were prespecified as negative controls for the interaction analysis, for which Ω was expected not to depart from 1 (Table S4).

#### 2.5.8. Competition and Masking

Because lower-than-expected reporting can arise from reporting artifacts, two further analyses were performed for PTs with lower-than-expected reporting [12,13]. To address event competition, that is, under-reporting of CRS, ICANS, or cytopenias in combination reports because reporters focus on the infections that dominate them, the analysis was repeated after excluding from all cohorts the reports containing any infection-related PT, identified from a list of infection terms approximating the System Organ Class “Infections and infestations”. To address masking by drugs strongly associated with these events, the analysis was repeated after removing from the background all reports listing other T-cell-engaging bispecific antibodies or chimeric antigen receptor (CAR) T-cell products (blinatumomab, glofitamab, epcoritamab, mosunetuzumab, tarlatamab, tebentafusp, elranatamab, talquetamab, linvoseltamab, axicabtagene ciloleucel, tisagenlecleucel, lisocabtagene maraleucel, brexucabtagene autoleucel, idecabtagene vicleucel, ciltacabtagene autoleucel, and obecabtagene autoleucel).

### 2.6. Statistical Considerations, Reporting, and Ethics

Consistent with the hypothesis-generating nature of disproportionality methods, no adjustment for multiple comparisons was applied [6,7]. With 79 PTs for which Ω was estimable at a two-sided α of 0.05, approximately four classifications could arise by chance alone; accordingly, statistical significance should not be interpreted as evidence of causality or clinical incidence. Statistical and clinical significance were distinguished throughout. Statistical significance refers only to the confidence-interval criteria above and indicates a departure from the expected reporting pattern. Clinical relevance was judged separately by (i) the frequency of the event among combination reports, (ii) its usual severity, (iii) the robustness of the estimate across sensitivity analyses, and (iv) whether a clinical action is available; these judgments are summarized for the principal findings at the start of the Discussion section. The study is reported in accordance with the READUS-PV guideline for disproportionality analyses [14], and the completed checklist is provided in the Supplementary Tables S1–S7. Detailed analytic procedures are provided in the Supplementary Methods. This study used only de-identified, publicly available FAERS data and did not involve human participants, identifiable private information, or interventions; therefore, it did not constitute human-subjects research and was exempt from institutional review board review and the requirement for informed consent.

## 3. Results

### 3.1. Reporting Cohorts

The analysis included 22,357 FAERS reports involving at least one of the two drugs of interest: 353 Tec-Dara combination reports, 2774 teclistamab-only reports, and 19,230 daratumumab-only reports against a background of 4,610,008 other FAERS reports (Table 1). Because the study window closed before the combination was approved, none of the 353 combination reports reflects use of the approved regimen. Nearly all combination reports (345; 97.7%) were expedited manufacturer reports compared with 71.0% of teclistamab-only and 76.0% of daratumumab-only reports, and 43 (12.2%) were literature-based; the public FAERS extracts do not identify clinical-study reports. Of the combination reports, 251 (71.1%) were submitted by health professionals, 292 (82.7%) originated outside the United States, and 143 (40.5%) were received in 2024–2025; the earliest combination report was first received in Q4 2021 and entered the study window through a later version. Both agents were coded as suspect (PS/SS) in 327 (92.6%) combination reports, only one agent in 13 (3.7%), and neither in 13 (3.7%); 216 (61.2%) combination reports also listed an immunomodulatory drug or proteasome inhibitor. A total of 102 MedDRA Preferred Terms met the prespecified reporting threshold in the combination cohort; Ω could be estimated for 79, of which 27 showed higher-than-expected reporting, 18 showed lower-than-expected reporting, and 34 showed no departure from the multiplicative expectation (all estimates in Table S2). Among the PTs of clinical interest (Table 3), the interaction profile had three components, described in turn below: higher-than-expected reporting of pneumonia and influenza; a divergent hematologic pattern, with lower-than-expected reporting of lineage-specific cytopenias but higher-than-expected reporting of bone marrow failure, based on few reports; and lower-than-expected reporting of the immune effector and humoral toxicities that characterize teclistamab monotherapy.

#### 3.1.1. Demographics and Reporting Trends

The median age was 68 years (IQR, 62–72) in the combination cohort, compared with 69 years in both the teclistamab-only and daratumumab-only cohorts; 36.0%, 29.6%, and 35.9% of reports, respectively, concerned women (Table 1). Age was missing in 9.3% and sex in 5.9% of combination reports, compared with 53.6% and 37.0% of teclistamab-only reports. Combination reports accrued early, with 36.8% received by the end of Q1 2023 and a peak in 2023; only 40.5% were received within the contemporaneous window (Figure 1). Teclistamab-only reports rose over the first two quarters after approval and remained stable thereafter, not consistent with a Weber effect, whereas daratumumab-only reports were stable across the study period apart from a transient decrease in Q3 2025. Four (1.1%) combination reports were received after the MajesTEC-3 results were first presented (December 2025), so notoriety-driven reporting is unlikely to have influenced the combination cohort.

#### 3.1.2. Method Validity

Positive controls behaved as expected: labeled reactions of teclistamab were strongly disproportionate in the teclistamab-only cohort (CRS, ROR 149; ICANS, ROR 148; hypogammaglobulinemia, ROR 42.5), as were labeled cytopenias of daratumumab in the daratumumab-only cohort (neutropenia, ROR 7.6; thrombocytopenia, ROR 6.8) (Table 3). Of the negative controls, musculoskeletal pain and headache were reported in fewer than three combination reports, so Ω could not be estimated; nausea showed higher-than-expected (Ω, 2.59; 95% CI, 1.31–5.11) and fatigue lower-than-expected reporting (Ω, 0.42; 95% CI, 0.19–0.90) (Table S4). Departures of this size for events without a plausible pharmacological interaction indicate that Ω is also shaped by reporting patterns, and they reinforce a cautious interpretation of the interaction findings.

### 3.2. Infections: Excess Reporting Concentrated in the Respiratory Tract

Infections were the most frequently reported adverse-event category with the combination, occurring in 197 out of 353 (55.8%) Tec-Dara reports, compared with 29.9% of teclistamab-only and 22.9% of daratumumab-only reports (Table 2 and Table S1). The interaction analysis showed that this excess was not distributed evenly across infection types (Table 3). Pneumonia, the most frequently reported infection (*n* = 57; ROR, 11.7; 95% CI, 8.8–15.5), had reporting odds approximately 1.5 times those expected from the individual agents (Ω, 1.50; 95% CI, 1.06–2.12), and influenza was also reported more often than expected (Ω, 2.63; 95% CI, 1.20–5.77). Respiratory tract infection (Ω, 1.47; 95% CI, 0.77–2.81) and fungal pneumonia (Ω, 4.12; 95% CI, 0.84–20.2) did not depart from the multiplicative expectation, although both exceeded expectation on the additive scale (Section 3.6). Bronchitis (Ω, 3.41; 95% CI, 0.94–12.3) rests on four combination reports and did not depart from expectation.

In contrast, infections that were already frequently reported with each agent alone were not reported more often than expected with the combination. Sepsis (Ω, 0.89; 95% CI, 0.54–1.47) and COVID-19 pneumonia (Ω, 0.77; 95% CI, 0.40–1.47) were disproportionately reported in all three cohorts (single-agent RORs, 3.2–8.8), but their combination RORs were consistent with the multiplicative expectation, although both exceeded expectation on the additive scale (Section 3.6). The infection findings therefore indicate a respiratory-predominant excess, most consistent for pneumonia, rather than a uniform increase across all infections.

### 3.3. Hematologic and Immune-Effector Toxicities

The hematologic findings moved in opposite directions: lineage-specific cytopenias were reported less often than expected, whereas a small number of reports of bone marrow failure exceeded expectation. Neutropenia (Ω, 0.16; 95% CI, 0.10–0.27), thrombocytopenia (Ω, 0.16; 95% CI, 0.08–0.32), and anemia (Ω, 0.14; 95% CI, 0.06–0.36) each showed lower-than-expected reporting, and febrile neutropenia did not depart from expectation (Ω, 1.44; 95% CI, 0.82–2.50). Bone marrow failure had a large but highly imprecise interaction ratio (Ω, 13.9; 95% CI, 4.02–48.3) that should be regarded as a hypothesis-generating outlier: it rests on 13 combination, 4 teclistamab-only, and 14 daratumumab-only reports, depends on fewer than five reports in one cohort, and could not be estimated in the contemporaneous window. This divergence may reflect reporters describing combination-treated patients with a single composite term for multilineage cytopenia, a genuinely more prolonged multilineage suppression with the combination or both; FAERS cannot distinguish these explanations (Section 4).

The toxicities that characterize teclistamab monotherapy were not amplified by the combination. Cytokine release syndrome (CRS; Ω, 0.15; 95% CI, 0.11–0.21), immune effector cell-associated neurotoxicity syndrome (ICANS; Ω, 0.11; 95% CI, 0.05–0.26), and hypogammaglobulinemia (Ω, 0.08; 95% CI, 0.04–0.16) were all reported less often than expected. Each was already very strongly associated with teclistamab alone (single-agent RORs, 149, 148, and 42.5, respectively), so the multiplicative expectation for the combination was correspondingly high. These results indicate that adding daratumumab did not increase the reporting of teclistamab’s hallmark toxicities; as discussed in Section 4, they should not be interpreted as a protective effect. Against a myeloma-restricted background, CRS and ICANS no longer departed from expectation (Section 3.6).

### 3.4. Reported Death Outcomes

A death outcome was reported in 55 out of 353 (15.6%) Tec-Dara reports compared with 625 out of 2774 (22.5%) teclistamab-only reports and 1960 out of 19,230 (10.2%) daratumumab-only reports. Death as a MedDRA PT was coded in only two combination reports, below the prespecified threshold, and was therefore not evaluated in the interaction analysis (Table S5). The lower proportion of fatal outcomes in combination than in teclistamab-only reports is consistent with differences in the treated populations: the combination was studied in patients with one to three prior lines of therapy, whereas teclistamab monotherapy was used after four or more prior lines. Because fatal outcomes in FAERS are not necessarily attributable to the reported drugs and depend strongly on line of therapy and disease stage, this finding should not be interpreted as a difference in survival.

### 3.5. Contemporaneous-Window Sensitivity Analysis

In the prespecified sensitivity analysis restricted to reports first received in Q1 2024–Q4 2025 (143 combination, 1786 teclistamab-only, and 10,816 daratumumab-only reports), the pneumonia estimate was similar in magnitude to that of the primary analysis (Ω, 1.68; 95% CI, 1.00–2.82), whereas respiratory tract infection (Ω, 1.97; 95% CI, 0.88–4.41) and influenza (Ω, 2.44; 95% CI, 0.79–7.55) were not statistically significant (Table 4 and Table S3). Lower-than-expected reporting of neutropenia, thrombocytopenia, hypogammaglobulinemia, CRS, and ICANS was preserved. Ω for bone marrow failure could not be calculated because the teclistamab-only count fell below the prespecified reporting threshold, so the interaction signal for this term could not be confirmed in the restricted window. Because fewer than half of the combination reports fell within this window, it provides a less precise test of the primary findings.

### 3.6. Sparse-Count, Drug-Role, and Comparator Sensitivity Analyses

#### 3.6.1. Sparse Counts

For the PTs in Table 3, the Haldane–Anscombe correction changed only the classification of bronchitis, whose lower confidence limit moved just above 1 (Table 5). With the shrinkage Ω of Norén et al., higher-than-expected reporting was retained for pneumonia (Ω_N_, 1.19; Ω_025_, 0.81), influenza (1.28; 0.34), and bone marrow failure (3.58; 2.79), and emerged for respiratory tract infection (1.82; 1.15) and fungal pneumonia (2.12; 0.70), but not for bronchitis (1.02; −0.40). Fungal pneumonia, bronchitis, and bone marrow failure each had fewer than five reports in at least one cohort. Lower-than-expected reporting of neutropenia, thrombocytopenia, anemia, CRS, and ICANS persisted under the additive model, whereas hypogammaglobulinemia no longer differed from expectation. Sepsis, COVID-19 pneumonia, and febrile neutropenia, which did not depart from the multiplicative expectation, showed higher-than-expected reporting under the additive model, illustrating the scale dependence of interaction classifications. Across the 79 PTs with estimable Ω, 76 classifications were unchanged by the Haldane–Anscombe correction and 47 were concordant with the shrinkage Ω (Table S6).

#### 3.6.2. Drug Role and Comparator

When exposure was restricted to suspect roles (327 combination, 2718 teclistamab-only, and 15,222 daratumumab-only reports), higher-than-expected reporting was retained for pneumonia (Ω, 1.62; 95% CI, 1.14–2.30) and influenza (Ω, 3.17; 95% CI, 1.38–7.26), and respiratory tract infection remained non-significant (Ω, 1.36; 95% CI, 0.67–2.73). With the background restricted to 139,684 myeloma reports, RORs were attenuated as expected (pneumonia ROR, 4.9 vs. 11.7), whereas Ω for pneumonia increased to 3.59 (95% CI, 2.54–5.07), and respiratory tract infection (Ω, 2.86; 95% CI, 1.48–5.52) and influenza (Ω, 3.89; 95% CI, 1.77–8.57) were also higher than expected; against this background, CRS (Ω, 1.03) and ICANS (Ω, 0.65) no longer departed from expectation, and the estimates for neutropenia (Ω, 0.54) and thrombocytopenia (Ω, 0.49) moved toward 1. Excluding reports that co-listed an immunomodulatory drug or proteasome inhibitor left 137 combination reports; Ω for pneumonia was of similar magnitude but no longer statistically significant (1.53; 95% CI, 0.85–2.74), and most other PTs could not be estimated (Table S7).

#### 3.6.3. Competition and Masking

After the exclusion of infection-containing reports from all cohorts, Ω was 0.14 (95% CI, 0.09–0.23) for CRS and 0.11 (95% CI, 0.03–0.36) for neutropenia; ICANS and thrombocytopenia could not be estimated (two combination reports each). Lower-than-expected reporting therefore persisted. Removing CAR T-cell and other bispecific antibody reports from the background further lowered the estimates for CRS (Ω, 0.03) and ICANS (Ω, 0.01) (Table S7) and did not materially change those for the cytopenias.

#### 3.6.4. Report Source

Because the public FAERS extracts do not identify clinical-study reports, the interaction analysis could not be stratified by reporting channel (Section 2.5).

#### 3.6.5. Treatment Population

Within the myeloma-restricted population (*n* = 162,041), after adjustment for age group, sex, year of initial receipt, report type, reporter region, and co-reported drug classes, the adjusted Ω was 3.08 (95% CI, 2.16–4.39) for pneumonia, 2.67 (95% CI, 1.35–5.29) for respiratory tract infection, and 2.84 (95% CI, 1.28–6.33) for influenza. With the daratumumab comparator restricted to relapsed-disease regimens (4278 reports), Ω for pneumonia was 1.43 (95% CI, 0.99–2.07), similar in magnitude but no longer statistically significant, whereas influenza remained higher than expected (Ω, 2.49; 95% CI, 1.07–5.75) (Table S7).

## 4. Discussion

This pharmacovigilance analysis identified a distinct adverse event reporting profile for the Tec-Dara combination, characterized by higher-than-expected reporting of respiratory infections, principally pneumonia and influenza, with no evidence of higher-than-expected reporting for common cytopenias or immune effector toxicities. To our knowledge, this is the first large-scale pharmacovigilance study to quantitatively evaluate drug–drug interaction signals associated with the teclistamab–daratumumab combination using a formal interaction framework. It provides pathogen-specific details that complement the aggregate infection rates reported in registration trials. Unlike previous studies describing the frequency of infectious adverse events with BCMA-directed therapies [15], the present study specifically quantified whether teclistamab plus daratumumab generated adverse-event reporting beyond that expected from the individual agents using a formal drug–drug interaction framework.

Table 6 summarizes the principal findings and separates their statistical strength from their clinical relevance. Only the pneumonia signal combines statistical evidence that was consistent in direction across all sensitivity analyses with high clinical relevance, although it lost statistical significance when reports co-listing an immunomodulatory drug or proteasome inhibitor were excluded and when the daratumumab comparator was restricted to relapsed-disease regimens. Influenza was also reported more often than expected but rests on few reports. The remaining findings are statistically fragile (bone marrow failure), scale dependent (respiratory tract infection, fungal pneumonia, sepsis, COVID-19 pneumonia, and hypogammaglobulinemia), not robust (bronchitis), or reflect reporting patterns without a direct clinical implication (lineage-specific cytopenias, CRS, and ICANS). The paragraphs that follow address these groups in that order.

A biological interaction is one plausible explanation for the respiratory signal. Teclistamab drives BCMA-directed T-cell activation, resulting in hypogammaglobulinemia and progressive lymphocyte exhaustion [16]. Daratumumab depletes CD38^+^ natural killer cells as well as immunosuppressive CD38^+^ regulatory T cells and myeloid-derived suppressor cells [17]. Combined humoral and cellular immune dysfunction could impair host defense against respiratory pathogens and may contribute to the respiratory-predominant reporting pattern observed in this analysis, although these mechanisms remain hypotheses that the present data cannot test.

Non-biological explanations must be weighed against this interpretation, and we rank them by their likely influence. First, and most important, is the reporting channel. Because all combination reports predate approval, many are likely to derive from clinical studies that capture low-grade respiratory infections systematically, whereas single-agent reports are more often spontaneous and weighted toward serious events; this could inflate the combination reporting of common respiratory terms. The effect is unlikely to account for the whole signal because trial capture should apply equally to sepsis, COVID-19 pneumonia, CRS, and cytopenias, none of which showed higher-than-expected reporting on the multiplicative scale, and because the signal included pneumonia, a predominantly serious event. The public FAERS extracts do not identify clinical-study reports, so this explanation could not be tested directly; nearly all combination reports (97.7%) were expedited manufacturer reports, which is compatible with, but does not demonstrate, a trial-derived component. Second is chance. With 79 PTs for which Ω was estimable, approximately four classifications could be spurious at a two-sided α of 0.05, and both estimable negative controls departed from 1 (nausea, Ω 2.59; fatigue, Ω 0.42) without a plausible pharmacological basis. Chance or non-specific reporting differences are less likely for pneumonia (57 reports; higher than expected under both the multiplicative and the shrinkage estimator) but plausible for influenza (9 reports) considered individually, and respiratory tract infection and fungal pneumonia exceeded expectation only on the additive scale. Third, calendar and seasonal confounding, including the Weber effect, is possible, because combination reports accrued over only a few respiratory seasons and were concentrated in 2023, before the contemporaneous window. The quarterly accumulation of reports (Figure 1) does not show an early post-launch peak in teclistamab-only reporting, which rose over the first two quarters after approval and then remained stable. In the contemporaneous window, the pneumonia estimate was similar in magnitude (Ω 1.68; 95% CI, 1.00–2.82) but less precise, and the other respiratory terms were not statistically significant; a temporal artifact therefore cannot be excluded, although the pneumonia estimate did not diminish. Fourth, residual confounding by treatment population is a major concern. The determinants of infection risk (line of therapy, disease burden, prior therapies, age, and baseline immunoglobulin levels) are unequally distributed across the cohorts and largely unrecorded in FAERS, and their effects on Ω are not uniform. The teclistamab-only cohort, drawn from heavily pretreated patients, raises ROR_tec_ and biases Ω downward. The daratumumab-only cohort, which includes patients with newly diagnosed disease, lowers ROR_dara_ and biases Ω upward, and could therefore generate an apparent interaction without any drug–drug interaction. Protocol-mandated immunoglobulin replacement and prophylaxis in combination-treated trial patients would bias toward the null. The analyses designed to address this gave divergent results. After covariate adjustment within the myeloma population, the interaction for pneumonia was larger rather than smaller (adjusted Ω 3.08; 95% CI, 2.16–4.39), and respiratory tract infection (2.67; 1.35–5.29) and influenza (2.84; 1.28–6.33) also exceeded expectation. In contrast, with the daratumumab comparator restricted to relapsed-disease regimens (Ω 1.43; 95% CI, 0.99–2.07) and after exclusion of reports co-listing an immunomodulatory drug or proteasome inhibitor (Ω 1.53; 95% CI, 0.85–2.74), the pneumonia estimate was of similar magnitude but no longer statistically significant; the latter analysis retained only 137 combination reports. Confounding by unrecorded factors, including line of therapy and partner agents, therefore cannot be excluded. On balance, we consider a genuine increase in respiratory infections with the combination a plausible explanation for the pneumonia signal, which was consistent in direction across all sensitivity analyses, while acknowledging that differential reporting and residual confounding by treatment population may account for part or all of its magnitude.

For bone marrow failure, the ranking is reversed. The estimate rests on 13 combination and only 4 teclistamab-only reports, the teclistamab-only count fell below the threshold in the contemporaneous window, and the concurrent lower-than-expected reporting of lineage-specific cytopenias suggests term selection by reporters. Chance and coding practice are therefore at least as likely as a biological interaction, and the signal should be regarded as a hypothesis for prospective evaluation.

The lower-than-expected reporting of CRS, ICANS, isolated cytopenias, and hypogammaglobulinemia requires particular caution, because several reporting mechanisms can produce Ω < 1 without any change in underlying risk [10]. First, these are labeled and very frequent toxicities of teclistamab, so the single-agent RORs are large and the multiplicative expectation becomes correspondingly demanding: for CRS, Ω = 1 would have required more than half (56%) of all combination reports to mention CRS, whereas 16% did. Second, many combination reports are likely to arise from clinical studies, in which expected protocol-managed events such as step-up-dose CRS are captured in trial databases but may be less often the focus of an individual safety report than unexpected or serious events such as infections; conversely, CRS and neurotoxicity, which carry a boxed warning for teclistamab, are likely to be preferentially reported in spontaneous postmarketing reports of teclistamab monotherapy (notoriety bias), whereas in combination reports, they may be overshadowed by infections (competition bias). Third, the combination was studied in patients with one to three prior lines of therapy, whereas teclistamab monotherapy was used in heavily pretreated, often triple-class-refractory disease with greater tumor burden, which itself increases the risk of CRS and cytopenias. Lower-than-expected reporting of CRS, ICANS, and isolated cytopenias persisted under the additive shrinkage model (Section 3.6), so it is not solely an artifact of the multiplicative scale, but remains a statement about reporting rather than risk. In addition, when the background was restricted to myeloma reports, CRS (Ω 1.03) and ICANS (Ω 0.65) no longer departed from expectation and the cytopenia estimates moved toward 1, indicating that much of the apparent deficit reflects the contrast with a largely non-oncology background rather than a property of the combination. These findings therefore indicate only that the combination was not reported with these toxicities more often than expected; they should not be interpreted as evidence that daratumumab protects against immune effector toxicity, cytopenias, or hypogammaglobulinemia.

We considered masking and competition biases explicitly, because they determine whether Ω < 1 could represent true antagonism [12,13,18]. Masking by drugs in the background, such as CAR T-cell products and other bispecific antibodies that contribute many CRS and ICANS reports, cannot explain these findings. Because Ω = odds_combo_ × odds_background_/(odds_tec_ × odds_dara_), the background odds enter Ω once, in the numerator, and removing masking drugs lowers them and moves Ω further below 1, as confirmed in the sensitivity analysis (CRS Ω 0.03; ICANS Ω 0.01; Section 3.6). Event competition within reports is more plausible: infections were recorded in 56% of combination reports, and if their prominence displaced CRS, ICANS, or cytopenia terms from those reports, Ω would be biased downward; excluding infection-containing reports did not materially change these estimates (Section 3.6). Drug competition may also act in the teclistamab-only cohort: as boxed-warning toxicities of teclistamab, CRS and neurotoxicity are likely to be preferentially reported with teclistamab monotherapy, which inflates ROR_tec_ and hence the multiplicative expectation. Consistent with recent guidance that inverse disproportionality signals describe lower-than-expected reporting rather than protection [18], we attribute the lower-than-expected reporting of CRS, ICANS, and cytopenias to these reporting mechanisms together with differences in the treated populations, and we do not claim true pharmacological antagonism. Determining whether daratumumab modifies these toxicities would require a comparison of incidence in trials or cohorts with defined exposure, with adjustment for line of therapy.

These results align with those of MajesTEC-3, in which severe infections clustered within the first six months and improved after protocol amendments that reinforced IgG-agnostic IVIG replacement and stringent antimicrobial prophylaxis [2]. In the MajesTEC-2 and TRIMM-2 studies, six out of seven deaths were infection-related and occurred before the implementation of intensified infection-prevention measures [19]. While MajesTEC-3 established a high overall burden of infectious complications, our findings extend these observations by suggesting that higher-than-expected reporting is concentrated among respiratory infections, principally pneumonia and influenza, rather than uniformly across all infectious complications. These findings support the continued emphasis on proactive infection prevention strategies, including antimicrobial prophylaxis, vaccination, immunoglobulin replacement when clinically indicated, and vigilant respiratory infection surveillance in patients receiving BCMA-directed combinations [4,5].

From a myeloma practice perspective, these findings point to three priorities, which should be regarded as hypotheses for prospective evaluation rather than as practice-changing evidence. First, the respiratory predominance of higher-than-expected reporting reinforces consensus recommendations on infection prevention in multiple myeloma and during bispecific antibody therapy, which address immunoglobulin replacement, antimicrobial and antiviral prophylaxis, and vaccination [20,21], and it supports a low threshold for respiratory viral testing and the early evaluation of pneumonia in patients receiving Tec-Dara, particularly during respiratory-virus seasons. Second, the bone marrow failure signal, although sparse, identifies prolonged or trilineage cytopenia as a toxicity domain that merits systematic capture, including time to count recovery, in ongoing and future combination trials. Third, because the regimen is now used earlier in the disease course, where the balance between durable disease control and cumulative infection risk differs from that in heavily pretreated disease, approaches that may reduce cumulative immunosuppression, such as less frequent dosing after response, deserve prospective evaluation with the combination. More broadly, this analysis illustrates how interaction-focused pharmacovigilance can complement registration trials during the early adoption of novel combination regimens in oncology, when trial populations are small and selected. These priorities are translated into numbered recommendations, each labeled by its evidentiary basis, in Table 7.

**Table 7:** Practical recommendations for patients receiving teclistamab plus daratumumab, with their evidentiary basis.

Basis	Recommendation	#
This study (higher-than-expected pneumonia and influenza reporting); consensus for PCR panels and treatment holds [22]	Evaluate any new respiratory symptom or fever the same day with a multiplex respiratory pathogen PCR panel and chest imaging; withhold Tec-Dara during active infection until clinical resolution.	1
Trial protocol [2]; consensus [22]; prescribing information [23]; not informed by the present data	Measure serum IgG at baseline and monthly. Give subcutaneous or intravenous immunoglobulin to maintain serum IgG > 400 mg/dL, as required by the prescribing information; replacement regardless of IgG level, as in the amended MajesTEC-3 protocol, may be considered.	2
Consensus [22]	*Pneumocystis* prophylaxis for all patients with trimethoprim–sulfamethoxazole (dapsone or atovaquone if intolerant) throughout treatment.	3
Consensus [22]; prescribing information recommends considering antiviral prophylaxis [23]	Herpes simplex/varicella zoster prophylaxis with acyclovir or valacyclovir throughout treatment.	4
Consensus [20,22]; influenza among the higher-than-expected signals in this study	Annual influenza vaccination (high-dose vaccine or two-dose series), COVID-19 vaccination per current guidance, and pneumococcal and non-live zoster vaccines; avoid live vaccines.	5
Consensus [22]	No routine antibacterial or antifungal prophylaxis; consider levofloxacin during prolonged neutropenia (ANC < 0.5 × 10^9^/L). For pneumonia not responding to antibacterial therapy, obtain chest CT and fungal diagnostics early.	6
Prescribing information [23] and consensus [22]; biopsy threshold proposed by the authors, adapted from late ICAHT [24]; not based on the bone marrow failure signal in this study	Withhold Tec-Dara for ANC < 0.5 × 10^9^/L or febrile neutropenia; use G-CSF for grade ≥ 3 neutropenia outside the CRS risk period. Perform bone marrow aspirate and biopsy when grade ≥ 3 cytopenia in ≥2 lineages persists > 30 days after the last dose despite G-CSF, once other causes have been excluded.	7

Items 1 and 5 draw on signals from this study, which are hypothesis-generating; the remaining items restate existing consensus guidance so that the table can serve as a practical checklist. None has been validated prospectively for Tec-Dara. ANC, absolute neutrophil count; CRS, cytokine release syndrome; G-CSF, granulocyte colony-stimulating factor; ICAHT, immune effector cell-associated hematotoxicity; PCR, polymerase chain reaction.

The March 2026 approval has changed the regulatory and practice context in which these signals should be read. The combination received traditional approval for patients who have received at least one prior line of therapy, including a proteasome inhibitor and an immunomodulatory agent [3]. This indication has no upper limit on prior lines, unlike MajesTEC-3, which enrolled patients with one to three prior lines, so practice populations may include more heavily pretreated patients than those who generated most combination reports in this study. The updated prescribing information retains the boxed warning for CRS and neurologic toxicity and the restricted TECVAYLI and TALVEY Risk Evaluation and Mitigation Strategy (REMS), which requires prescriber and pharmacy certification and patient wallet cards. It specifies 48-h hospitalization after each of the first two step-up doses and a de-escalating schedule: weekly to week 8, every two weeks to week 24, and monthly thereafter [23,25]. For infections, the label recommends monitoring immunoglobulin levels before and during treatment with replacement to maintain serum IgG above 400 mg/dL, antimicrobial prophylaxis according to guidelines, consideration of antiviral prophylaxis against herpes zoster, and withholding treatment for grade ≥ 3 infections [23]. Hypogammaglobulinemia was reported in 84% of combination-treated patients in the registration trial [23], a reminder that the lower-than-expected reporting observed here does not indicate low incidence. The REMS, however, is directed at CRS and neurologic toxicity, whereas both MajesTEC-3 and the present analysis identify infection, particularly respiratory infection, as the dominant toxicity of the combination. Two priorities for postmarketing surveillance follow. First, this analysis should be repeated once post-approval FAERS quarters are available to test whether the respiratory signal persists under label-directed immunoglobulin replacement, prophylaxis, and the de-escalating schedule. Second, infection-specific measures, such as the documentation of immunoglobulin monitoring and prophylaxis, merit consideration in risk-management materials for the combination.

As discussed above, the bone marrow failure signal (Ω = 13.9) is imprecise, with a confidence interval spanning an order of magnitude, and may partly reflect term selection. Persistent multilineage cytopenia during Tec-Dara therapy should be evaluated as for any patient receiving T-cell-engaging therapy (Table 7, item 7); the present signal does not by itself justify a different approach. More broadly, lower-than-expected reporting for isolated cytopenias, CRS, ICANS, and hypogammaglobulinemia likely reflects differences in background reporting frequencies, treatment settings, disease characteristics, exposure duration, or other reporting biases rather than protective biological interactions.

This study has several limitations. First, spontaneous reporting is voluntary, incomplete, and potentially biased; FAERS lacks exposure denominators, precluding the estimation of true incidence or relative risk; the Tec-Dara cohort remains small (*n* = 353); and temporal confounding is inherent to disproportionality analysis. Second, the study window ended before approval of the combination, so many combination reports are likely to derive from clinical studies with systematic adverse-event collection and protocol-mandated prophylaxis, whereas single-agent reports are more often spontaneous; this difference in reporting channel may affect which events are reported and could bias Ω in either direction; because the public FAERS extracts do not identify clinical-study reports, this could not be examined directly. Third, confounding by indication is likely: the combination cohort comprises patients with one to three prior lines of therapy, the teclistamab-only cohort predominantly heavily pretreated patients, and the daratumumab-only cohort patients across all lines, including newly diagnosed disease. Because these differences act in opposite directions on Ω, lowering the daratumumab-only reference risk while raising the teclistamab-only reference risk, they could produce an apparent interaction independently of a true drug–drug interaction. The covariate-adjusted and relapsed-disease comparator analyses reduce, but cannot eliminate, this concern, because FAERS does not record the line of therapy, disease burden, or immunoglobulin levels. The full-database background further differs from all three cohorts in underlying disease; the myeloma-restricted comparator reduces, but does not eliminate, this imbalance. Fourth, co-reporting of both agents does not establish concurrent administration; co-reported partner drugs (immunomodulatory drugs, proteasome inhibitors, and corticosteroids) could contribute to the observed events, and some duplicate reports (e.g., a single trial event submitted by more than one source) may persist despite deduplication. Fifth, Ω measures departure from a multiplicative expectation; interaction classifications are scale dependent, and under the additive shrinkage Ω, the classifications of sepsis, COVID-19 pneumonia, febrile neutropenia, hypogammaglobulinemia, respiratory tract infection, and fungal pneumonia changed, whereas those of pneumonia, influenza, bronchitis, bone marrow failure, isolated cytopenias, CRS, and ICANS did not. In addition, infection-containing reports in the competition analysis were identified from a list of infection-related Preferred Terms rather than the full MedDRA System Organ Class. Sixth, because FAERS contains neither exposure denominators nor efficacy outcomes, this study characterizes only the toxicity side of the benefit–risk balance and cannot estimate a therapeutic index or net clinical benefit. The survival advantage of Tec-Dara in MajesTEC-3 [2] should frame the interpretation of these signals, which indicate where supportive care can be targeted rather than whether the regimen should be used. Seventh, patient-level clinical information, including disease status, prior therapies, concomitant medications, immunoglobulin levels, antimicrobial prophylaxis, and treatment duration, was unavailable, limiting the adjustment for potential confounding factors. Finally, spontaneous reporting systems cannot establish the temporality or causality of adverse events.

Although the large FAERS background dataset, the positive-control results, the contemporaneous-window, sparse-count, drug-role, and myeloma-restricted sensitivity analyses, and the consistency of signals across related Preferred Terms improve signal robustness, they do not eliminate the inherent limitations of spontaneous reporting analyses. Disproportionality findings are hypothesis-generating and require confirmation in prospective, denominator-based cohort studies.

As BCMA- and CD38-directed combinations move into earlier lines of therapy, prospective pharmacovigilance and real-world cohort studies incorporating denominator-based analyses are needed to determine whether these interaction signals translate into clinically meaningful toxicities.

## 5. Conclusions

These findings identify a distinct pattern of higher-than-expected respiratory infection reporting associated with the teclistamab–daratumumab combination, principally pneumonia and influenza, whereas CRS and ICANS were not reported more often than expected. Although these findings should not be interpreted as evidence of causality or clinical incidence, and the pneumonia signal lost statistical significance in two out of the four analyses addressing confounding by treatment population, they support one concrete practice: in patients receiving Tec-Dara, any new respiratory symptom or fever should prompt same-day evaluation, including multiplex respiratory pathogen testing and chest imaging, in addition to guideline-recommended immunoglobulin replacement, antimicrobial prophylaxis, and vaccination [20,21]. A numbered set of practical recommendations, each labeled by its evidentiary basis, is provided in Table 7.

The bone marrow failure signal is a statistically fragile, hypothesis-generating outlier and should not inform practice until it is confirmed. Prospective denominator-based studies are needed to validate both the respiratory infection interaction signal and the bone marrow failure signal observed in this analysis.

## Figures and Tables

**Table 1:** Cohort overview, demographics, report characteristics, and aggregate event rates among teclistamab- and daratumumab-related FAERS reports, Q4 2022–Q4 2025.

Background (*n* = 4,610,008)	Dara Only (*n* = 19,230)	Tec Only (*n* = 2774)	Tec-Dara (*n* = 353)	Variable
—	4407 (22.9)	830 (29.9)	197 (55.8)	Infection reports, *n* (%)
—	3542 (18.4)	328 (11.8)	78 (22.1)	Cytopenia reports, *n* (%)
361,459 (7.8)	1960 (10.2)	625 (22.5)	55 (15.6)	Death outcome, *n* (%)
**Demographics**				
59 (41–71)	69 (60–76)	69 (60–76)	68 (62–72)	Age, median (IQR), years
1,034,035 (22.4)	8901 (46.3)	837 (30.2)	206 (58.4)	Age ≥ 65 years, *n* (%)
1,966,933 (42.7)	5118 (26.6)	1488 (53.6)	33 (9.3)	Age missing, *n* (%)
2,269,201 (49.2)	6898 (35.9)	821 (29.6)	127 (36.0)	Female, *n* (%)
791,887 (17.2)	3718 (19.3)	1027 (37.0)	21 (5.9)	Sex missing, *n* (%)
**Report characteristics**				
2,462,497 (53.4)	14,618 (76.0)	1970 (71.0)	345 (97.7)	Expedited (15-day) manufacturer reports, *n* (%)
1,948,911 (42.3)	3877 (20.2)	677 (24.4)	8 (2.3)	Periodic manufacturer reports, *n* (%)
198,600 (4.3)	735 (3.8)	127 (4.6)	0 (0.0)	Direct (voluntary) reports, *n* (%)
397,604 (8.6)	2126 (11.1)	139 (5.0)	43 (12.2)	Literature-based reports, *n* (%)
1,252,859 (27.2)	10,273 (53.4)	1724 (62.1)	251 (71.1)	Submitted by a health professional, *n* (%)
1,482,511 (32.2)	12,541 (65.2)	1186 (42.8)	292 (82.7)	Reports from outside the United States, *n* (%)
2,602,207 (56.4)	10,816 (56.2)	1786 (64.4)	143 (40.5)	Received in 2024–2025, *n* (%)
—	15,222 (79.2)	2718 (98.0)	327 (92.6)	Drug(s) of interest coded as suspect (PS/SS), *n* (%)

Infection reports, reports with ≥1 PT in the MedDRA System Organ Class “Infections and infestations”; cytopenia reports, reports with ≥1 cytopenia-related PT (Section 2.2); percentages are of reports in each cohort, and a report can contribute to more than one category. Death outcome, OUTC code DE. Age was converted to years from AGE and AGE_COD. Report type is from REPT_COD; literature-based reports have a non-empty LIT_REF; health professional, OCCP_COD MD, PH, or OT; outside the United States, OCCR_COUNTRY (REPORTER_COUNTRY when missing) other than the U.S.; receipt year, INIT_FDA_DT. Suspect role: both agents coded PS or SS (Tec-Dara) or the agent of interest coded PS or SS (single-agent cohorts). The public FAERS extracts do not identify clinical-study reports. The study window (Q4 2022–Q4 2025) closed before the March 2026 approval of the combination, so no combination report reflects use of the approved regimen. PS, primary suspect; SS, secondary suspect. Abbreviations: Dara, daratumumab; FAERS, U.S. Food and Drug Administration Adverse Event Reporting System; Tec, teclistamab.

**Table 2:** Two-by-two contingency table constructed for each exposure cohort and each MedDRA Preferred Term (PT).

Total	Reports Without the PT	Reports with the PT	
a + b	b	a	Exposure cohort (combination, teclistamab-only, or daratumumab-only)
c + d	d	c	Background (reports listing neither agent)

ROR = (a/b)/(c/d); PRR = [a/(a + b)]/[c/(c + d)]; SE(ln ROR) = √(1/a + 1/b + 1/c + 1/d). For the interaction ratio, Ω = ROR_combo_/(ROR_tec_ × ROR_dara_), with Var(ln Ω) = Σ(1/a_k_ + 1/b_k_) over the three exposure cohorts + 1/c + 1/d. The background cells c and d are identical for the three exposure cohorts. PRR, proportional reporting ratio; ROR, reporting odds ratio; SE, standard error.

**Table 3:** Disproportionality and Ω interaction estimates for the teclistamab–daratumumab combination, FAERS Q4 2022–Q4 2025.

Interaction		Dara Only (*n* = 19,230)		Tec Only (*n* = 2774)		Tec-Dara (*n* = 353)		Adverse Event (MedDRA PT)
Reporting	Ω (95% CI)	ROR (95% CI)	*n*	ROR (95% CI)	*n*	ROR (95% CI)	*n*	
**Infections**								
Higher	1.50 (1.06–2.12)	2.9 (2.7–3.1)	872	2.7 (2.2–3.2)	118	11.7 (8.8–15.5)	57	Pneumonia
NS	1.47 (0.77–2.81)	4.4 (3.7–5.2)	130	5.4 (3.6–8.1)	23	34.6 (21.5–55.6)	18	Respiratory tract infection
Higher	2.63 (1.20–5.77)	1.1 (0.9–1.3)	149	1.3 (0.9–1.9)	25	3.7 (1.9–7.1)	9	Influenza
NS	3.41 (0.94–12.3)	1.6 (1.3–1.9)	94	0.7 (0.3–1.5)	6	3.6 (1.4–9.7)	4	Bronchitis
NS	4.12 (0.84–20.2)	2.6 (1.5–4.3)	14	3.8 (1.2–11.9)	3	40.4 (15.0–108)	4	Fungal pneumonia
NS	0.89 (0.54–1.47)	3.2 (2.8–3.5)	290	5.1 (4.0–6.5)	67	14.4 (9.4–21.9)	23	Sepsis
NS	0.77 (0.40–1.47)	8.8 (7.5–10.2)	171	7.8 (5.1–11.9)	22	52.5 (32.7–84.5)	18	COVID-19 pneumonia
**Hematologic toxicity**								
Higher	13.9 (4.02–48.3)	1.9 (1.1–3.2)	14	3.8 (1.4–10.0)	4	99.7 (57.2–174)	13	Bone marrow failure
NS	1.44 (0.82–2.50)	5.4 (4.9–6.0)	374	2.8 (1.9–4.1)	28	21.8 (14.6–32.5)	26	Febrile neutropenia
Lower	0.16 (0.10–0.27)	7.6 (7.1–8.0)	1076	5.7 (4.7–6.8)	118	6.9 (4.3–11.0)	18	Neutropenia
Lower	0.16 (0.08–0.32)	6.8 (6.3–7.3)	667	4.6 (3.6–5.9)	66	4.9 (2.6–9.6)	9	Thrombocytopenia
Lower	0.14 (0.06–0.36)	4.1 (3.7–4.4)	606	3.0 (2.4–3.9)	66	1.8 (0.7–4.3)	5	Anemia
Lower	0.08 (0.04–0.16)	22.0 (19.0–25.5)	192	42.5 (32.3–55.9)	53	76.7 (43.1–137)	12	Hypogammaglobulinemia
**Immune effector toxicity**								
Lower	0.15 (0.11–0.21)	4.0 (3.4–4.6)	163	149 (136–162)	674	89.2 (67.1–118)	57	Cytokine release syndrome
Lower	0.11 (0.05–0.26)	1.7 (1.1–2.5)	24	148 (130–168)	279	26.8 (12.7–56.7)	7	ICANS

Abbreviations: CI, confidence interval; ICANS, immune effector cell-associated neurotoxicity syndrome; NS, not statistically significant; PT, MedDRA Preferred Term; ROR, reporting odds ratio; Ω, multiplicative interaction ratio, ROR_combo_/(ROR_tec_ × ROR_dara_). Higher, higher-than-expected reporting (lower 95% CI of Ω > 1); Lower, lower-than-expected reporting (upper 95% CI of Ω < 1); NS, 95% CI of Ω includes 1. “Lower” denotes a reporting pattern and does not imply reduced risk. Each ROR contrasts the indicated cohort with the complete FAERS background (Table 2); PRRs for every PT are provided in Table S2. The MedDRA PT “Death” (2 combination reports) did not meet the ≥3-report threshold and is not shown; death outcomes are summarized in Table 1. Bone marrow failure rests on fewer than five teclistamab-only reports and could not be estimated in the contemporaneous window; it is shown as a hypothesis-generating outlier. *n*, number of reports in each cohort that included the PT.

**Table 4:** Sensitivity analysis: Ω in the contemporaneous window (Q1 2024–Q4 2025) versus the full study period.

Δ	Ω, Sensitivity (95% CI)	*n* Combo, Sens. Window	Primary Signal	Ω, Primary (95% CI)	Preferred Term
↑	1.68 (1.00–2.82)	23	Higher	1.50 (1.06–2.12)	Pneumonia
↔	1.97 (0.88–4.41)	11	NS	1.47 (0.77–2.81)	Respiratory tract infection
Lost	2.44 (0.79–7.55)	4	Higher	2.63 (1.20–5.77)	Influenza
↔	0.31 (0.09–1.04)	3	NS	0.89 (0.54–1.47)	Sepsis
—	Not computable *	3	Higher	13.9 (4.02–48.3)	Bone marrow failure
—	Not computable †	2	NS	1.44 (0.82–2.50)	Febrile neutropenia
↔	0.22 (0.12–0.40)	13	Lower	0.16 (0.10–0.27)	Neutropenia
↔	0.35 (0.16–0.76)	8	Lower	0.16 (0.08–0.32)	Thrombocytopenia
↔	0.10 (0.05–0.21)	11	Lower	0.08 (0.04–0.16)	Hypogammaglobulinemia
↔	0.21 (0.14–0.33)	35	Lower	0.15 (0.11–0.21)	Cytokine release syndrome
↔	0.23 (0.09–0.60)	6	Lower	0.11 (0.05–0.26)	ICANS

* Bone marrow failure Ω was not computable in the restricted window because the teclistamab-only count fell below the prespecified 3-report threshold. † Febrile neutropenia Ω was not computable because the combination count fell below the threshold. The window was defined by the date of initial FDA receipt. Δ denotes the change from the primary to the sensitivity analysis: ↑, larger Ω with the same classification; ↔, same classification; Lost, statistical significance lost; —, not evaluable. Abbreviations are as listed in Table 3.

**Table 5:** Robustness of interaction classifications to sparse counts and to the interaction scale.

Classification	≥5 Reports per Cohort	Ω_N_ (95% Interval)	Ω, Haldane–Anscombe (95% CI)	Ω, Primary (95% CI)	Preferred Term
Concordant	Yes	1.19 (0.81 to 1.56)	1.51 (1.07–2.12)	1.50 (1.06–2.12)	Pneumonia
Scale dependent	Yes	1.82 (1.15 to 2.49)	1.47 (0.77–2.79)	1.47 (0.77–2.81)	Respiratory tract infection
Concordant	Yes	1.28 (0.34 to 2.22)	2.70 (1.25–5.84)	2.63 (1.20–5.77)	Influenza
Not robust	No	1.02 (−0.40 to 2.43)	3.52 (1.03–12.0)	3.41 (0.94–12.3)	Bronchitis
Scale dependent; sparse	No	2.12 (0.70 to 3.53)	3.84 (0.86–17.1)	4.12 (0.84–20.2)	Fungal pneumonia
Scale dependent	Yes	0.91 (0.32 to 1.50)	0.90 (0.55–1.48)	0.89 (0.54–1.47)	Sepsis
Scale dependent	Yes	1.61 (0.95 to 2.28)	0.77 (0.40–1.46)	0.77 (0.40–1.47)	COVID-19 pneumonia
Concordant; sparse	No	3.58 (2.79 to 4.36)	12.4 (3.77–40.8)	13.9 (4.02–48.3)	Bone marrow failure
Scale dependent	Yes	1.47 (0.91 to 2.02)	1.43 (0.83–2.49)	1.44 (0.82–2.50)	Febrile neutropenia
Concordant	Yes	−0.76 (−1.43 to −0.10)	0.16 (0.10–0.27)	0.16 (0.10–0.27)	Neutropenia
Concordant	Yes	−0.99 (−1.94 to −0.05)	0.17 (0.08–0.33)	0.16 (0.08–0.32)	Thrombocytopenia
Concordant	Yes	−1.62 (−2.89 to −0.36)	0.16 (0.07–0.38)	0.14 (0.06–0.36)	Anemia
Scale dependent	Yes	0.25 (−0.56 to 1.07)	0.08 (0.04–0.16)	0.08 (0.04–0.16)	Hypogammaglobulinemia
Concordant	Yes	−0.61 (−0.98 to −0.23)	0.15 (0.11–0.21)	0.15 (0.11–0.21)	Cytokine release syndrome
Concordant	Yes	−2.27 (−3.34 to −1.20)	0.11 (0.05–0.26)	0.11 (0.05–0.26)	ICANS

Ω_N_, shrinkage Ω of Norén et al. (log_2_ scale; additive model), with its 95% interval (Ω_025_ to Ω_975_); Ω_025_ > 0 indicates higher-than-expected and Ω_975_ < 0 lower-than-expected reporting. Concordant, same classification under all methods; sparse, fewer than five reports in at least one exposure cohort; scale dependent, classification differs between the multiplicative (Ω) and additive (Ω_N_) models; not robust, classification differs between the continuity-corrected and the uncorrected or shrinkage estimates. Abbreviations are as listed in Table 3.

**Table 6:** Principal findings: statistical strength versus clinical relevance.

Interpretation	Clinical Relevance	Statistical Evidence	Finding
Principal finding; supports respiratory-focused prevention (Table 7, items 1 and 5)	High: pneumonia in 16% of combination reports; frequently serious; preventable and treatable	Ω 1.50 (95% CI, 1.06–2.12); 57 reports; higher than expected on the additive scale; retained with suspect roles only (Ω 1.62), a myeloma background (Ω 3.59), and covariate adjustment (Ω 3.08); similar in magnitude but not significant after excluding IMiD/PI co-listing (Ω 1.53; 0.85–2.74) or with a relapsed-disease daratumumab comparator (Ω 1.43; 0.99–2.07)	Pneumonia
Consistent with the respiratory pattern; individually imprecise or scale dependent	Potentially high (influenza is vaccine-preventable; fungal pneumonia is often severe), but few reports	Influenza Ω 2.63 (95% CI, 1.20–5.77), 9 reports; respiratory tract infection (Ω 1.47; 0.77–2.81; 18 reports) and fungal pneumonia (Ω 4.12; 0.84–20.2; 4 reports) higher than expected on the additive scale only	Influenza; respiratory tract infection; fungal pneumonia
Not interpreted	Low: usually mild	Ω 3.41 (95% CI, 0.94–12.3); not robust to continuity correction; 4 reports	Bronchitis
Frequently reported with each agent alone; scale dependent	High severity, but no consistent evidence of interaction	No departure on the multiplicative scale; higher than expected on the additive scale	Sepsis; COVID-19 pneumonia
Hypothesis-generating outlier	Potentially high severity; not actionable on current evidence	Ω 13.9 (95% CI, 4.02–48.3); 4 teclistamab-only reports; not estimable in the contemporaneous window	Bone marrow failure
Reporting pattern; no change to monitoring	Limited: cytopenias remain very common with the combination; lower reporting does not mean lower incidence	Lower than expected on both scales against the full background; attenuated against a myeloma background (neutropenia Ω 0.54; thrombocytopenia Ω 0.49)	Neutropenia; thrombocytopenia; anemia
Reporting pattern; not protective	Reassuring only in that the combination did not amplify reporting; boxed-warning toxicities remain	Lower than expected on both scales against the full background (Ω 0.15 and 0.11); no departure from expectation against a myeloma background (Ω 1.03 and 0.65)	CRS; ICANS
Scale dependent; replace per label (Table 7, item 2)	High: very frequent with the combination, and immunoglobulin replacement is required by the label	Lower than expected on the multiplicative scale only	Hypogammaglobulinemia

Statistical evidence refers to the interaction ratio Ω and its robustness (Table 3 and Table 5); clinical relevance was judged from event frequency, usual severity, robustness, and actionability (Section 2.6). A statistically significant Ω indicates a departure from the expected reporting pattern and implies neither clinical importance nor causality. CRS, cytokine release syndrome; IMiD, immunomodulatory drug; PI, proteasome inhibitor. Other abbreviations are as listed in Table 3.

## Data Availability

FAERS data are publicly available at https://fis.fda.gov/extensions/FPD-QDE-FAERS/FPD-QDE-FAERS.html (accessed on 8 May 2026). The drug-name search terms are given in Section 2.2, and derived cohort-level counts and estimates are provided in the manuscript and Supplementary Materials; the analytic code is available from the corresponding author on reasonable request.

## Supplementary Materials

The following supporting information can be downloaded at: https://www.mdpi.com/article/10.3390/cancers18203388/s1, Supplementary Methods: Detailed methods, operational definitions, and worked calculation example; Table S1: Cohort overview and aggregate event rates, FAERS Q4 2022–Q4 2025; Table S2: Disproportionality (ROR and PRR) and Ω interaction estimates for all Preferred Terms meeting the reporting threshold, FAERS Q4 2022–Q4 2025; Table S3: Contemporaneous-window sensitivity analysis (reports first received Q1 2024–Q4 2025): cohort sizes and estimates for the Preferred Terms of clinical interest; Table S4: Positive- and negative-control Preferred Terms, FAERS Q4 2022–Q4 2025; Table S5: Death outcome by exposure cohort; Table S6: Sparse-count sensitivity analyses for all Preferred Terms with estimable Ω: Haldane–Anscombe-corrected Ω, shrinkage Ω (additive model), and ≥5-report restriction; Table S7: Drug-role, myeloma-restricted comparator, partner-agent-exclusion, covariate-adjusted, relapsed-disease comparator, competition, and masking analyses.
