# Supplementary material for: Infectious Complications and Toxicities of the Teclistamab Plus Daratumumab Combination in Relapsed or Refractory Multiple Myeloma: A Disproportionality and Drug–Interaction Analysis of the FDA Adverse Event Reporting System
Source: Cancers (Basel). 2026 Oct 20;18(20):3388. doi: 10.3390/cancers18203388 (PMC13702562; doi:10.3390/cancers18203388)
Supplement: Supplementary file 1 [file cancers-18-03388-s001.zip › cancers-4549326-supplementary.pdf]

Supplementary Materials

Infectious Complications and Toxicities of the Teclistamab plus Daratumumab Combination in Relapsed or Refractory Multiple Myeloma: A Disproportionality and Drug–Interaction Analysis of the FDA Adverse Event Reporting System

Carmel Awadallah, Sohaib Irfan, Al-Ola Abdallah, and Muhammad Bilal Abid

Contents

- Supplementary Methods: Detailed methods, operational definitions, and worked calculation example.
- Table S1. Cohort overview and aggregate event rates, FAERS Q4 2022–Q4 2025.
- Table S2. Disproportionality (ROR and PRR) and Ω interaction estimates for all Preferred Terms meeting the reporting threshold, FAERS Q4 2022–Q4 2025.
- Table S3. Contemporaneous-window sensitivity analysis (reports first received Q1 2024–Q4 2025): cohort sizes and estimates for the Preferred Terms of clinical interest.
- Table S4. Positive- and negative-control Preferred Terms, FAERS Q4 2022–Q4 2025.
- Table S5. Death outcome by exposure cohort.
- Table S6. Sparse-count sensitivity analyses for all Preferred Terms with estimable Ω: Haldane–Anscombe-corrected Ω, shrinkage Ω (additive model), and ⩾5-report restriction.
- Table S7. Drug-role, myeloma-restricted comparator, partner-agent-exclusion, covariate-adjusted, relapsed-disease comparator, competition, and masking analyses.

Supplementary Methods

S1. Data source, study period, and deduplication

FAERS quarterly data extract files (DEMO, DRUG, REAC, OUTC, RPSR, and INDI) from Q4 2022 to Q4 2025 (13 quarters) were obtained from https://fis.fda.gov/extensions/FPD-QDE-FAERS/FPD-QDE-FAERS.html. The window starts with the quarter of the accelerated approval of teclistamab (25 October 2022) and ends with the last complete calendar quarter before FDA approval of the teclistamab–daratumumab combination (5 March 2026); all combination reports therefore describe pre-approval exposure. Deduplication followed FDA recommendations: across all 13 quarters combined, only the most recent version of each CASEID was retained (latest FDA_DT, with ties broken by the highest case version), and each retained report was linked to the DRUG, REAC, OUTC, RPSR, and INDI records of that version. This yielded 4,632,365 unique cases.

S2. Drug-name harmonization and exposure cohorts

Drug entries were identified in both DRUGNAME and PROD_AI by case-insensitive text matching of "teclistamab", "Tecvayli", and "teclistamab-cqyv" for teclistamab and "daratumumab", "Darzalex" (which also captures Darzalex Faspro), and "hyaluronidase-fihj" for daratumumab. Intravenous and subcutaneous daratumumab were pooled. Within each report, multiple entries for the same agent were collapsed into one exposure indicator and duplicate PTs were collapsed. In the primary analysis, a drug counted as reported in any role (PS, SS, C, or I). Four mutually exclusive report-level cohorts were defined: combination (both agents; n = 353), teclistamab-only (n = 2,774), daratumumab-only (n = 19,230), and background (neither agent; n = 4,610,008). "Combination" denotes co-reporting within a single case report; concurrent administration cannot be verified in FAERS.

S3. Operational definitions

- **Adverse events:** MedDRA Preferred Terms (version 29.0) from REAC, without a priori restriction; PTs reported in fewer than three combination reports were not analyzed.
- **Infection reports (Table 2, Table S1):** reports with at least one PT in the System Organ Class "Infections and infestations".
- **Infection-containing reports (competition analysis):** reports with at least one PT from an infection-related Preferred Term, identified by text matching of Preferred Term names (e.g., pneumonia, sepsis, bacteraemia, viral, fungal, and bacterial infections, COVID-19, influenza, herpes zoster, cytomegalovirus, and Pneumocystis jirovecii infection).
- **Cytopenia reports (Table 2, Table S1):** reports with at least one of the PTs neutropenia, febrile neutropenia, thrombocytopenia, anemia, leukopenia, or pancytopenia.
- **Bone marrow failure:** the MedDRA PT of that name, analyzed separately from the lineage-specific cytopenia PTs.
- **Death outcome:** OUTC code DE. The MedDRA PT "Death" (two combination reports) did not meet the reporting threshold.
- **Report characteristics:** report type from REPT_COD (EXP, PER, DIR); literature-based reports, non-empty LIT_REF; health professional, OCCP_COD MD, PH, or OT; reporter country, OCCR_COUNTRY (REPORTER_COUNTRY when missing); receipt date, INIT_FDA_DT; age converted to years from AGE and AGE_COD.

S4. Disproportionality analysis

For each exposure cohort and each PT, a 2 × 2 table was constructed against the background cohort (main text, Table 1), with (a) the cohort reports with the PT; (b) the cohort reports without it; (c) the background reports with the PT; and (d) the background reports without it.

$$\mathrm{ROR} = (a/b)/(c/d); \quad \mathrm{PRR} = [a/(a + b)]/[c/(c + d)]$$

$$\mathrm{SE}(\ln \mathrm{ROR}) = \sqrt{1/a + 1/b + 1/c + 1/d}; \quad 95\%\ \mathrm{CI} = \exp(\ln \mathrm{ROR} \pm 1.96 \times \mathrm{SE}) \quad \text{(logit/Woolf method)}$$

A PT was considered disproportionately reported when $a \geq 3$ and the lower 95% confidence limit of the ROR exceeded 1.

S5. Drug–drug interaction (Ω)

Interaction was quantified with the multiplicative model of Thakrar et al. [1], reviewed by Noguchi et al. [2]:

$$\Omega = \mathrm{ROR}_{combo}/(\mathrm{ROR}_{tec} \times \mathrm{ROR}_{dara})$$

Because $\ln \Omega = \mathrm{logit}(p_{combo}) - \mathrm{logit}(p_{tec}) - \mathrm{logit}(p_{dara}) + \mathrm{logit}(p_{background})$ and the four cohorts are disjoint:

$$\mathrm{Var}(\ln \Omega) = 1/a_{combo} + 1/b_{combo} + 1/a_{tec} + 1/b_{tec} + 1/a_{dara} + 1/b_{dara} + 1/c + 1/d; \quad 95\%\ \mathrm{CI} = \exp(\ln \Omega \pm 1.96 \times \sqrt{\mathrm{Var}})$$

Ω was computed for PTs with at least three reports in each exposure cohort. A lower 95% confidence limit above 1 was classified as higher-than-expected reporting, an upper limit below 1 as lower-than-expected reporting, and an interval including 1 as no departure from the multiplicative expectation. These classifications describe reporting frequency, not risk.

S6. Worked example: pneumonia

The counts below reproduce the pneumonia row of main-text Table 3.

Cohort	With pneumonia	Without pneumonia	Total	ROR (95% CI)
Tec-Dara (combination)	a = 57	b = 296	353	11.7 (8.8–15.5)
Teclistamab-only	a = 118	b = 2,656	2,774	2.7 (2.2–3.2)
Daratumumab-only	a = 872	b = 18,358	19,230	2.9 (2.7–3.1)
Background	c = 74,641	d = 4,535,367	4,610,008	—

- Point estimate: Ω = 11.7/(2.7 × 2.9) = 1.49 with the rounded RORs shown, and 1.50 with unrounded values.
- Variance of ln Ω from the exposure cohorts: 1/57 + 1/296 + 1/118 + 1/2,656 + 1/872 + 1/18,358 = 0.03097. The background terms add 1/74,641 + 1/4,535,367 = 0.00001. Var(ln Ω) = 0.03099; SE = 0.176.
- 95% CI: exp(ln 1.50 ± 1.96 × 0.176) = 1.06 to 2.12, i.e., Ω 1.50 (95% CI, 1.06–2.12): higher-than-expected reporting.
- Interpretation: the combination's reporting odds for pneumonia were about 1.5 times those expected if the associations of the two agents with pneumonia combined multiplicatively.

S7. Sensitivity analyses

- **Contemporaneous window:** all cohorts, including the background, restricted to reports first received (INIT_FDA_DT) in Q1 2024–Q4 2025.

- **Sparse counts:** (i) the shrinkage Ω of Norén et al. [3], $\Omega_N = \log_2[(n_{111} + 0.5)/(E_{111} + 0.5)]$, with $E_{111} = n_{11} \times g_{11}$ and $g_{11} = 1 - 1/[\max(o_{00}, o_{10}) + \max(o_{00}, o_{01}) - o_{00} + 1]$, where o_{00}, o_{10}, and o_{01} are the event odds in the background, teclistamab-only, and daratumumab-only cohorts, and $\Omega_{025}/\Omega_{975} = \Omega_N \mp 1.96/(\ln 2 \times \sqrt{n_{111}})$; (ii) the Haldane–Anscombe correction (0.5 added to every cell); and (iii) restriction to PTs with at least five reports in each exposure cohort.
- **Drug role:** combination restricted to reports in which both agents were coded PS or SS; single-agent cohorts to reports in which the agent of interest was coded PS or SS.
- **Myeloma-restricted background:** reports with an indication PT of "Plasma cell myeloma" (or related PTs containing "plasma cell myeloma" or "multiple myeloma") or listing lenalidomide, pomalidomide, thalidomide, bortezomib, carfilzomib, ixazomib, isatuximab, elotuzumab, elranatamab, talquetamab, linvoseltamab, idecabtagene vicleucel, ciltacabtagene autoleucel, belantamab mafodotin, selinexor, or melphalan flufenamide, and listing neither teclistamab nor daratumumab (139,684 reports).
- **Partner-agent exclusion:** reports co-listing lenalidomide, pomalidomide, thalidomide, bortezomib, carfilzomib, or ixazomib excluded from all exposure cohorts.
- **Covariate-adjusted Ω:** within the myeloma-restricted population ($n = 162{,}041$), logistic regression for each primary PT with indicators for teclistamab, daratumumab, and their product, adjusted for age group, sex, year of initial receipt, report type, reporter region, and co-reported immunomodulatory drugs, proteasome inhibitors, and corticosteroids; missing covariates were assigned to a missing-indicator category. The exponentiated interaction coefficient is the adjusted Ω.
- **Relapsed-disease daratumumab comparator:** daratumumab-only cohort restricted to reports co-listing pomalidomide or carfilzomib (4278 reports).
- **Positive and negative controls:** positive controls were labelled reactions of each agent (CRS, ICANS, and hypogammaglobulinemia for teclistamab; neutropenia and thrombocytopenia for daratumumab). Negative controls were four PTs not expected to be associated with either agent or their combination (musculoskeletal pain, headache, nausea, and fatigue), for which Ω was expected not to depart from 1.
- **Competition:** reports containing any infection-related PT (Section S3) excluded from all cohorts.
- **Masking:** reports listing blinatumomab, glofitamab, epcoritamab, mosunetuzumab, tarlatamab, tebentafusp, elranatamab, talquetamab, linvoseltamab, axicabtagene ciloleucel, tisagenlecleucel, lisocabtagene maraleucel, brexucabtagene autoleucel, idecabtagene vicleucel, ciltacabtagene autoleucel, or obecabtagene autoleucel removed from the background.

S8. Multiplicity

No adjustment for multiple comparisons was applied, consistent with the hypothesis-generating nature of disproportionality analysis. With 79 PTs for which Ω was estimable, about four classifications would be expected to arise by chance at a two-sided α of 0.05.

References

1. Thakrar, B.T.; Grundschober, S.B.; Doessegger, L. Detecting signals of drug–drug interactions in a spontaneous reports database. Br. J. Clin. Pharmacol. 2007, 64, 489–495.

2. Noguchi, Y.; Tachi, T.; Teramachi, H. Review of statistical methodologies for detecting drug–drug interactions using spontaneous reporting systems. Front. Pharmacol. 2019, 10, 1319.

3. Norén, G.N.; Sundberg, R.; Bate, A.; Edwards, I.R. A statistical methodology for drug–drug interaction surveillance. Stat. Med. 2008, 27, 3057–3070.

Table S1. Cohort overview and aggregate event rates, FAERS Q4 2022–Q4 2025.

Variable	Tec-Dara (n = 353)	Tec only (n = 2,774)	Dara only (n = 19,230)	Background (N = 4,610,008)
Infection reports, n (%)	197 (55.8)	830 (29.9)	4407 (22.9)	—
Cytopenia reports, n (%)	78 (22.1)	328 (11.8)	3542 (18.4)	—
Death outcome, n (%)	55 (15.6)	625 (22.5)	1960 (10.2)	361,459 (7.8)
Age, median (IQR), years	68 (62–72)	69 (60–76)	69 (60–76)	59 (41–71)
Age ≥ 65 years, n (%)	206 (58.4)	837 (30.2)	8901 (46.3)	1,034,035 (22.4)
Female, n (%)	127 (36.0)	821 (29.6)	6898 (35.9)	2,269,201 (49.2)
Received in 2024–2025, n (%)	143 (40.5)	1786 (64.4)	10,816 (56.2)	2,602,207 (56.4)

Infection reports, reports with ≥1 PT in the MedDRA System Organ Class "Infections and infestations"; cytopenia reports, reports with ≥1 of the PTs neutropenia, febrile neutropenia, thrombocytopenia, anemia, leukopenia, or pancytopenia; death outcome, OUTC code DE. A report can contribute to more than one category; —, not calculated for the background. Full demographic and report characteristics, including missing data, are given in main-text Table 2. Dara, daratumumab; IQR, interquartile range; Tec, teclistamab.

Table S2. Disproportionality (ROR and PRR) and Ω interaction estimates for all Preferred Terms meeting the reporting threshold, FAERS Q4 2022–Q4 2025.

Preferred Term	Tec-Dara (n = 353)			Tec only (n = 2774)			Dara only (n = 19,230)			Interaction	
	n	ROR (95% CI)	PRR	n	ROR (95% CI)	PRR	n	ROR (95% CI)	PRR	Ω (95% CI)	Reporting
Infection-related terms											
Bacterial infection	3	8.2 (2.6–25.5)	8.1	17	5.9 (3.7–9.5)	5.9	41	2.0 (1.5–2.8)	2.0	0.68 (0.19–2.43)	NS
Bacterial sepsis	11	216 (118–395)	209	4	9.7 (3.6–25.9)	9.7	26	9.1 (6.1–13.4)	9.1	2.45 (0.73–8.26)	NS
Bronchitis	4	3.6 (1.4–9.7)	3.6	6	0.7 (0.3–1.5)	0.7	94	1.6 (1.3–1.9)	1.5	3.41 (0.94–12.3)	NS
Colitis	4	5.0 (1.9–13.4)	4.9	29	4.6 (3.2–6.6)	4.6	49	1.1 (0.8–1.5)	1.1	0.98 (0.33–2.90)	NS
COVID-19	14	2.0 (1.2–3.4)	1.9	144	2.6 (2.2–3.1)	2.5	628	1.6 (1.5–1.7)	1.6	0.47 (0.27–0.83)	Lower
COVID-19 pneumonia	18	52.5 (32.7–84.5)	49.9	22	7.8 (5.1–11.9)	7.8	171	8.8 (7.5–10.2)	8.7	0.77 (0.40–1.47)	NS
Cytomegalovirus infection reactivation	7	40.5 (19.1–85.7)	39.7	23	16.7 (11.1–25.3)	16.6	101	10.6 (8.7–12.9)	10.5	0.23 (0.10–0.55)	Lower
Haemophilus infection	5	285 (117–697)	281	5	35.9 (14.8–87.1)	35.8	15	15.5 (9.2–26.2)	15.5	0.51 (0.13–1.98)	NS
Infection	12	4.3 (2.4–7.7)	4.2	146	6.8 (5.8–8.1)	6.5	454	3.0 (2.7–3.3)	2.9	0.21 (0.12–0.39)	Lower
Influenza	9	3.7 (1.9–7.1)	3.6	25	1.3 (0.9–1.9)	1.3	149	1.1 (0.9–1.3)	1.1	2.63 (1.20–5.77)	Higher
Pneumonia	57	11.7 (8.8–15.5)	10.0	118	2.7 (2.2–3.2)	2.6	872	2.9 (2.7–3.1)	2.8	1.50 (1.06–2.12)	Higher
Pneumonia bacterial	4	18.0 (6.7–48.2)	17.8	15	8.5 (5.1–14.2)	8.5	61	5.0 (3.9–6.4)	5.0	0.42 (0.14–1.32)	NS
Pneumonia fungal	4	40.4 (15.0–108)	39.9	3	3.8 (1.2–11.9)	3.8	14	2.6 (1.5–4.3)	2.6	4.12 (0.84–20.2)	NS
Respiratory tract infection	18	34.6 (21.5–55.6)	32.9	23	5.4 (3.6–8.1)	5.3	130	4.4 (3.7–5.2)	4.4	1.47 (0.77–2.81)	NS
Sepsis	23	14.4 (9.4–21.9)	13.5	67	5.1 (4.0–6.5)	5.0	290	3.2 (2.8–3.5)	3.1	0.89 (0.54–1.47)	NS
Septic shock	3	3.9 (1.2–12.1)	3.9	39	6.5 (4.7–8.9)	6.4	178	4.2 (3.6–4.9)	4.2	0.14 (0.04–0.47)	Lower
Staphylococcal bacteraemia	4	48.8 (18.2–131)	48.3	4	6.2 (2.3–16.4)	6.1	16	3.5 (2.2–5.8)	3.5	2.24 (0.51–9.79)	NS
Urinary tract infection	5	1.6 (0.7–3.8)	1.6	24	1.0 (0.6–1.4)	1.0	226	1.3 (1.1–1.5)	1.3	1.26 (0.47–3.35)	NS
Urosepsis	3	20.1 (6.5–62.7)	20.0	7	5.9 (2.8–12.5)	5.9	27	3.3 (2.3–4.8)	3.3	1.03 (0.25–4.20)	NS
Viral infection	3	4.6 (1.5–14.2)	4.5	15	2.9 (1.7–4.8)	2.9	48	1.3 (1.0–1.8)	1.3	1.19 (0.33–4.25)	NS
Hematologic and immune terms											
Anaemia	5	1.8 (0.7–4.3)	1.8	66	3.0 (2.4–3.9)	3.0	606	4.1 (3.7–4.4)	4.0	0.14 (0.06–0.36)	Lower
Bone marrow failure	13	99.7 (57.2–174)	96.0	4	3.8 (1.4–10.0)	3.8	14	1.9 (1.1–3.2)	1.9	13.9 (4.02–48.3)	Higher
Febrile neutropenia	26	21.8 (14.6–32.5)	20.2	28	2.8 (1.9–4.1)	2.8	374	5.4 (4.9–6.0)	5.3	1.44 (0.82–2.50)	NS
Hypogammaglobulinaemia	12	76.7 (43.1–137)	74.2	53	42.5 (32.3–55.9)	41.7	192	22.0 (19.0–25.5)	21.8	0.08 (0.04–0.16)	Lower
Neutropenia	18	6.9 (4.3–11.0)	6.6	118	5.7 (4.7–6.8)	5.5	1,076	7.6 (7.1–8.0)	7.2	0.16 (0.10–0.27)	Lower
Neutrophil count decreased	3	3.5 (1.1–10.9)	3.5	26	3.9 (2.6–5.7)	3.8	190	4.1 (3.5–4.7)	4.0	0.22 (0.07–0.74)	Lower
Thrombocytopenia	9	4.9 (2.6–9.6)	4.8	66	4.6 (3.6–5.9)	4.5	667	6.8 (6.3–7.3)	6.6	0.16 (0.08–0.32)	Lower
Other terms											
Abdominal pain	3	0.8 (0.3–2.4)	0.8	7	0.2 (0.1–0.5)	0.2	113	0.5 (0.4–0.6)	0.5	6.30 (1.60–24.8)	Higher
Acute kidney injury	3	1.0 (0.3–3.1)	1.0	31	1.3 (0.9–1.9)	1.3	296	1.8 (1.6–2.1)	1.8	0.41 (0.13–1.37)	NS
Acute myeloid leukaemia	6	23.9 (10.7–53.7)	23.5	3	1.5 (0.5–4.7)	1.5	52	3.8 (2.9–4.9)	3.7	4.26 (1.03–17.6)	Higher
Asthenia	7	1.1 (0.5–2.4)	1.1	37	0.8 (0.6–1.1)	0.8	413	1.2 (1.1–1.4)	1.2	1.20 (0.53–2.74)	NS
Condition aggravated	10	1.4 (0.7–2.6)	1.4	5	0.09 (0.04–0.2)	0.09	140	0.4 (0.3–0.4)	0.4	45.8 (15.4–136)	Higher
Cough	9	1.6 (0.8–3.2)	1.6	29	0.7 (0.5–1.0)	0.7	288	1.0 (0.8–1.1)	1.0	2.60 (1.21–5.59)	Higher
Cytokine release syndrome	57	89.2 (67.1–118)	74.9	674	149 (136–162)	113	163	4.0 (3.4–4.6)	3.9	0.15 (0.11–0.21)	Lower
Cytopenia	3	8.9 (2.8–27.6)	8.8	33	12.4 (8.8–17.6)	12.3	198	10.8 (9.3–12.4)	10.7	0.07 (0.02–0.22)	Lower

Preferred Term	Tec-Dara (n = 353)			Tec only (n = 2774)			Dara only (n = 19,230)			Interaction	
	n	ROR (95% CI)	PRR	n	ROR (95% CI)	PRR	n	ROR (95% CI)	PRR	Ω (95% CI)	Reporting
Decreased appetite	9	2.1 (1.1–4.1)	2.1	19	0.6 (0.4–0.9)	0.6	250	1.1 (0.9–1.2)	1.1	3.53 (1.57–7.94)	Higher
Depressed level of consciousness	20	37.3 (23.7–58.6)	35.2	9	2.0 (1.0–3.9)	2.0	17	0.5 (0.3–0.9)	0.5	33.6 (13.3–84.9)	Higher
Diarrhoea	12	1.0 (0.6–1.8)	1.0	67	0.7 (0.6–0.9)	0.7	860	1.4 (1.3–1.5)	1.4	1.04 (0.55–1.94)	NS
Disease progression	9	3.7 (1.9–7.2)	3.7	25	1.3 (0.9–1.9)	1.3	306	2.3 (2.1–2.6)	2.3	1.25 (0.57–2.71)	NS
Disseminated intravascular coagulation	3	18.2 (5.8–56.9)	18.1	3	2.3 (0.7–7.2)	2.3	17	1.9 (1.2–3.0)	1.9	4.21 (0.79–22.4)	NS
Dizziness	4	0.5 (0.2–1.4)	0.5	17	0.3 (0.2–0.4)	0.3	275	0.7 (0.6–0.7)	0.7	2.85 (0.95–8.57)	NS
Drug ineffective	21	0.9 (0.6–1.5)	0.9	111	0.6 (0.5–0.8)	0.6	953	0.8 (0.7–0.8)	0.8	1.94 (1.20–3.16)	Higher
Dyspnoea	10	1.1 (0.6–2.1)	1.1	34	0.5 (0.3–0.7)	0.5	598	1.2 (1.1–1.4)	1.2	1.88 (0.92–3.87)	NS
Fatigue	7	0.5 (0.2–1.0)	0.5	102	0.9 (0.7–1.1)	0.9	972	1.3 (1.2–1.4)	1.3	0.42 (0.19–0.90)	Lower
Gastrointestinal disorder	3	1.4 (0.4–4.4)	1.4	5	0.3 (0.1–0.7)	0.3	100	0.9 (0.7–1.0)	0.9	5.55 (1.30–23.7)	Higher
General physical health deterioration	23	9.8 (6.4–15.0)	9.3	17	0.9 (0.5–1.4)	0.9	124	0.9 (0.8–1.1)	0.9	12.3 (6.37–23.9)	Higher
Haemoptysis	4	10.7 (4.0–28.6)	10.6	3	1.0 (0.3–3.1)	1.0	20	1.0 (0.6–1.5)	1.0	10.9 (2.28–52.1)	Higher
Hyperhidrosis	3	1.6 (0.5–5.0)	1.6	6	0.4 (0.2–0.9)	0.4	70	0.7 (0.5–0.9)	0.7	5.81 (1.42–23.8)	Higher
Hypertension	22	6.4 (4.2–9.9)	6.1	11	0.4 (0.2–0.7)	0.4	189	1.0 (0.8–1.1)	1.0	17.4 (8.23–36.6)	Higher
Hypertransaminasaemia	4	15.9 (5.9–42.7)	15.8	7	3.5 (1.7–7.4)	3.5	10	0.7 (0.4–1.3)	0.7	6.27 (1.57–24.9)	Higher
Hypocalcaemia	4	12.8 (4.8–34.4)	12.7	4	1.6 (0.6–4.3)	1.6	68	4.0 (3.1–5.0)	4.0	2.00 (0.49–8.20)	NS
Hypokalaemia	4	5.3 (2.0–14.1)	5.2	15	2.5 (1.5–4.2)	2.5	123	3.0 (2.5–3.5)	2.9	0.71 (0.23–2.19)	NS
Immune effector cell-associated neurotoxicity syndrome	7	26.8 (12.7–56.7)	26.3	279	148 (130–168)	133	24	1.7 (1.1–2.5)	1.7	0.11 (0.05–0.26)	Lower
Intentional product use issue	8	3.3 (1.6–6.7)	3.3	19	1.0 (0.6–1.6)	1.0	748	5.8 (5.4–6.2)	5.6	0.58 (0.25–1.34)	NS
Lethargy	8	10.6 (5.3–21.4)	10.4	5	0.8 (0.3–2.0)	0.8	21	0.5 (0.3–0.8)	0.5	25.6 (7.71–85.3)	Higher
Loss of consciousness	3	1.7 (0.6–5.4)	1.7	4	0.3 (0.1–0.8)	0.3	72	0.8 (0.6–1.0)	0.8	7.87 (1.72–36.0)	Higher
Multiple organ dysfunction syndrome	20	30.4 (19.4–47.8)	28.8	8	1.5 (0.7–2.9)	1.5	90	2.4 (1.9–2.9)	2.4	8.71 (3.71–20.5)	Higher
Muscle spasms	3	1.1 (0.4–3.4)	1.1	6	0.3 (0.1–0.6)	0.3	160	1.1 (0.9–1.3)	1.1	3.66 (0.90–14.8)	NS
Nausea	11	0.9 (0.5–1.6)	0.9	40	0.4 (0.3–0.5)	0.4	584	0.9 (0.8–0.9)	0.9	2.59 (1.31–5.11)	Higher
Neuropathy peripheral	10	5.7 (3.0–10.7)	5.6	48	3.5 (2.6–4.6)	3.4	786	8.4 (7.8–9.0)	8.1	0.20 (0.10–0.40)	Lower
Off label use	16	0.6 (0.4–1.0)	0.6	177	0.9 (0.8–1.1)	0.9	2,118	1.6 (1.6–1.7)	1.6	0.42 (0.25–0.72)	Lower
Plasma cell myeloma	29	58.9 (40.3–86.2)	54.1	201	51.4 (44.4–59.5)	47.8	2,257	87.5 (83.3–92.0)	77.4	0.01 (0.01–0.02)	Lower
Plasma cell myeloma recurrent	9	132 (68.1–257)	129	3	5.5 (1.8–17.0)	5.5	182	48.3 (41.2–56.7)	47.9	0.50 (0.13–1.87)	NS
Plasma cell myeloma refractory	13	312 (178–547)	301	17	50.4 (31.1–81.8)	50.1	417	181 (159–206)	177	0.03 (0.02–0.07)	Lower
Plasmacytoma	13	881 (498–1560)	849	9	75.0 (38.4–146)	74.8	107	129 (102–163)	128	0.09 (0.04–0.22)	Lower
Product dose omission issue	3	0.3 (0.08–0.8)	0.3	43	0.5 (0.3–0.6)	0.5	313	0.5 (0.4–0.5)	0.5	1.12 (0.35–3.66)	NS
Product use in unapproved indication	4	0.6 (0.2–1.6)	0.6	5	0.09 (0.04–0.2)	0.09	129	0.3 (0.3–0.4)	0.4	18.2 (4.81–68.8)	Higher
Productive cough	4	3.9 (1.4–10.4)	3.8	6	0.7 (0.3–1.6)	0.7	60	1.1 (0.8–1.4)	1.1	4.99 (1.37–18.2)	Higher
Pruritus	5	0.6 (0.3–1.5)	0.6	10	0.2 (0.08–0.3)	0.2	181	0.4 (0.3–0.5)	0.4	9.89 (3.33–29.4)	Higher
Pulmonary oedema	20	33.2 (21.1–52.1)	31.4	5	1.0 (0.4–2.4)	1.0	72	2.1 (1.6–2.6)	2.1	16.0 (5.81–44.1)	Higher
Pyrexia	46	9.2 (6.8–12.6)	8.1	119	2.8 (2.3–3.3)	2.7	604	2.0 (1.8–2.2)	2.0	1.68 (1.16–2.43)	Higher
Rash	7	0.9 (0.4–1.9)	0.9	39	0.6 (0.5–0.9)	0.6	536	1.2 (1.1–1.4)	1.2	1.14 (0.50–2.57)	NS
Renal failure	21	15.0 (9.7–23.4)	14.2	23	2.0 (1.3–3.0)	2.0	170	2.1 (1.8–2.5)	2.1	3.57 (1.92–6.65)	Higher
Renal impairment	3	2.0 (0.6–6.1)	2.0	14	1.2 (0.7–2.0)	1.2	126	1.5 (1.3–1.8)	1.5	1.12 (0.32–3.96)	NS

Preferred Term	Tec-Dara (n = 353)			Tec only (n = 2774)			Dara only (n = 19,230)			Interaction	
	n	ROR (95% CI)	PRR	n	ROR (95% CI)	PRR	n	ROR (95% CI)	PRR	Ω (95% CI)	Reporting
Respiratory failure	3	3.0 (1.0–9.5)	3.0	18	2.3 (1.5–3.7)	2.3	159	3.0 (2.5–3.5)	2.9	0.44 (0.13–1.53)	NS
Tachycardia	20	15.1 (9.6–23.8)	14.3	18	1.6 (1.0–2.6)	1.6	84	1.1 (0.9–1.4)	1.1	8.31 (4.20–16.4)	Higher
Therapy non-responder	6	5.0 (2.2–11.2)	4.9	17	1.8 (1.1–2.9)	1.8	96	1.4 (1.2–1.8)	1.4	1.94 (0.74–5.06)	NS
Toxic skin eruption	4	26.5 (9.9–71.0)	26.2	5	4.2 (1.7–10.0)	4.2	35	4.2 (3.0–5.9)	4.2	1.51 (0.39–5.88)	NS
White blood cell count decreased	5	2.6 (1.1–6.3)	2.6	15	1.0 (0.6–1.6)	1.0	378	3.6 (3.3–4.0)	3.6	0.73 (0.26–2.02)	NS

The 79 Preferred Terms with at least three reports in each exposure cohort, for which Ω was estimable. Each ROR and PRR contrasts the indicated cohort with the background of reports listing neither agent (N = 4,610,008). Higher, lower 95% CI of Ω > 1; Lower, upper 95% CI of Ω < 1; NS, 95% CI includes 1. "Lower" denotes a reporting pattern and does not imply reduced risk. Preferred Terms are given as coded in MedDRA; grouping is descriptive. Dara, daratumumab; PRR, proportional reporting ratio; ROR, reporting odds ratio; Tec, teclistamab.

Table S2 (continued). Preferred Terms reported in at least three combination reports for which Ω was not estimable (fewer than three reports in a single-agent cohort).

Preferred Term	Tec-Dara, n	Tec only, n	Dara only, n
Ureteric haemorrhage	8	<3	<3
Device related sepsis	19	<3	9
Intervertebral discitis	14	<3	12
Aplasia	6	<3	<3
Invasive lobular breast carcinoma	3	<3	<3
Neutropenic infection	3	<3	5
Ureteric obstruction	4	<3	<3
Congenital aplasia	4	<3	3
Febrile bone marrow aplasia	12	<3	20
Thrombophlebitis	4	<3	17
Tachypnoea	20	<3	90
Abscess limb	6	<3	<3
Squamous cell carcinoma	7	<3	43
Diffuse large B-cell lymphoma	6	<3	8
White blood cell count increased	15	<3	15
Disturbance in attention	20	<3	17
Retching	8	<3	4
Haematuria	9	<3	17
Therapy partial responder	5	<3	168
Rhinorrhoea	8	<3	55
Rectal haemorrhage	3	<3	8
Rash pruritic	4	<3	70
Treatment failure	5	<3	52

<3, fewer than three reports. Ω requires at least three reports in each exposure cohort.

Table S3. Contemporaneous-window sensitivity analysis (reports first received Q1 2024–Q4 2025): cohort sizes and estimates for the Preferred Terms of clinical interest.

Preferred Term	Ω, primary (95% CI)	Primary	n combo, window	Ω, window (95% CI)	Δ
Pneumonia	1.50 (1.06–2.12)	Higher	23	1.68 (1.00–2.82)	↑
Respiratory tract infection	1.47 (0.77–2.81)	NS	11	1.97 (0.88–4.41)	↔
Influenza	2.63 (1.20–5.77)	Higher	4	2.44 (0.79–7.55)	Lost
Sepsis	0.89 (0.54–1.47)	NS	3	0.31 (0.09–1.04)	↔
Bone marrow failure	13.9 (4.02–48.3)	Higher	3	Not computable	—
Febrile neutropenia	1.44 (0.82–2.50)	NS	2	Not computable	—
Neutropenia	0.16 (0.10–0.27)	Lower	13	0.22 (0.12–0.40)	↔
Thrombocytopenia	0.16 (0.08–0.32)	Lower	8	0.35 (0.16–0.76)	↔
Hypogammaglobulinemia	0.08 (0.04–0.16)	Lower	11	0.10 (0.05–0.21)	↔
Cytokine release syndrome	0.15 (0.11–0.21)	Lower	35	0.21 (0.14–0.33)	↔
ICANS	0.11 (0.05–0.26)	Lower	6	0.23 (0.09–0.60)	↔

All cohorts, including the background, were restricted to reports first received by the FDA in Q1 2024–Q4 2025 (143 combination, 1786 teclistamab-only, and 10,816 daratumumab-only reports). Not computable: fewer than three reports in at least one exposure cohort (teclistamab-only for bone marrow failure; combination for febrile neutropenia). Δ: ↑, larger Ω with the same classification; ↔, same classification; Lost, statistical significance lost; —, not evaluable.

Table S4. Positive- and negative-control Preferred Terms, FAERS Q4 2022–Q4 2025.

Preferred Term	Cohort / measure	n	Estimate (95% CI)
Positive controls (labelled reactions; disproportionality in the single-agent cohort)			
Cytokine release syndrome	Teclistamab-only, ROR	674	149 (136–162)
ICANS	Teclistamab-only, ROR	279	148 (130–168)
Hypogammaglobulinemia	Teclistamab-only, ROR	53	42.5 (32.3–55.9)
Neutropenia	Daratumumab-only, ROR	1,076	7.6 (7.1–8.0)
Thrombocytopenia	Daratumumab-only, ROR	667	6.8 (6.3–7.3)
Negative controls (not expected to be associated with either agent; interaction ratio)			
Musculoskeletal pain	Combination, Ω	<3	Not estimable
Headache	Combination, Ω	<3	Not estimable
Nausea	Combination, Ω	11	2.59 (1.31–5.11)
Fatigue	Combination, Ω	7	0.42 (0.19–0.90)

Positive controls are expected to be strongly disproportionate in the cohort of the agent for which they are labelled; negative controls are expected to show Ω not different from 1. Musculoskeletal pain and headache were each reported in fewer than three combination reports (<3), so Ω could not be estimated; nausea and fatigue departed from 1 in opposite directions, indicating that Ω is also influenced by reporting patterns unrelated to pharmacology. Single-agent counts: nausea, 40 teclistamab-only and 584 daratumumab-only reports; fatigue, 102 and 972. n, number of reports with the PT in the indicated cohort (combination cohort for negative controls). ICANS, immune effector cell–associated neurotoxicity syndrome; ROR, reporting odds ratio; Ω, ROR_combo/(ROR_tec × ROR_dara).

Table S5. Death outcome by exposure cohort.

Cohort	Reports	Death outcome, n	% of reports
Tec-Dara combination	353	55	15.6
Teclistamab-only	2774	625	22.5
Daratumumab-only	19,230	1960	10.2
Background	4,610,008	361,459	7.8

Death outcome, OUTC code DE, irrespective of cause. The MedDRA PT "Death" was coded in two combination reports, below the ≥3-report threshold, and was not included in the interaction analysis. Fatal outcomes in FAERS are not necessarily attributable to the reported drugs and depend on line of therapy and disease stage; the percentages are not case-fatality rates and should not be compared as survival.

Table S6. Sparse-count sensitivity analyses for all Preferred Terms with estimable Ω: Haldane–Anscombe-corrected Ω, shrinkage Ω (additive model), and ≥5-report restriction.

Preferred Term	Ω, primary (95% CI)	Ω, Haldane–Anscombe (95% CI)	Ω_N (95% interval)	≥5 reports per cohort	Classification
Infection-related terms					
Bacterial infection	0.68 (0.19–2.43)	0.76 (0.23–2.51)	0.20 (−1.43 to 1.83)	No	Concordant; sparse
Bacterial sepsis	2.45 (0.73–8.26)	2.23 (0.70–7.14)	3.00 (2.15 to 3.86)	No	Scale dependent; sparse
Bronchitis	3.41 (0.94–12.3)	3.52 (1.03–12.0)	1.02 (−0.40 to 2.43)	No	Not robust
Colitis	0.98 (0.33–2.90)	1.07 (0.38–3.01)	0.07 (−1.34 to 1.49)	No	Concordant; sparse
COVID-19	0.47 (0.27–0.83)	0.48 (0.28–0.84)	−0.65 (−1.41 to 0.10)	Yes	Scale dependent
COVID-19 pneumonia	0.77 (0.40–1.47)	0.77 (0.40–1.46)	1.61 (0.95 to 2.28)	Yes	Scale dependent
Cytomegalovirus infection reactivation	0.23 (0.10–0.55)	0.24 (0.10–0.56)	0.56 (−0.51 to 1.63)	Yes	Scale dependent
Haemophilus infection	0.51 (0.13–1.98)	0.50 (0.14–1.81)	1.98 (0.72 to 3.25)	Yes	Scale dependent
Infection	0.21 (0.12–0.39)	0.22 (0.12–0.40)	−0.95 (−1.76 to −0.13)	Yes	Concordant
Influenza	2.63 (1.20–5.77)	2.70 (1.25–5.84)	1.28 (0.34 to 2.22)	Yes	Concordant
Pneumonia	1.50 (1.06–2.12)	1.51 (1.07–2.12)	1.19 (0.81 to 1.56)	Yes	Concordant
Pneumonia bacterial	0.42 (0.14–1.32)	0.46 (0.15–1.35)	0.45 (−0.96 to 1.86)	No	Concordant; sparse
Pneumonia fungal	4.12 (0.84–20.2)	3.84 (0.86–17.1)	2.12 (0.70 to 3.53)	No	Scale dependent; sparse
Respiratory tract infection	1.47 (0.77–2.81)	1.47 (0.77–2.79)	1.82 (1.15 to 2.49)	Yes	Scale dependent
Sepsis	0.89 (0.54–1.47)	0.90 (0.55–1.48)	0.91 (0.32 to 1.50)	Yes	Scale dependent
Septic shock	0.14 (0.04–0.47)	0.16 (0.05–0.49)	−1.17 (−2.81 to 0.46)	No	Scale dependent; sparse
Staphylococcal bacteraemia	2.24 (0.51–9.79)	2.17 (0.54–8.78)	1.88 (0.47 to 3.30)	No	Scale dependent; sparse
Urinary tract infection	1.26 (0.47–3.35)	1.35 (0.53–3.47)	0.24 (−1.02 to 1.51)	Yes	Concordant
Urosepsis	1.03 (0.25–4.20)	1.10 (0.29–4.14)	1.01 (−0.62 to 2.65)	No	Concordant; sparse
Viral infection	1.19 (0.33–4.25)	1.32 (0.40–4.39)	0.41 (−1.22 to 2.05)	No	Concordant; sparse
Hematologic and immune terms					
Anaemia	0.14 (0.06–0.36)	0.16 (0.07–0.38)	−1.62 (−2.89 to −0.36)	Yes	Concordant
Bone marrow failure	13.9 (4.02–48.3)	12.4 (3.77–40.8)	3.58 (2.79 to 4.36)	No	Concordant; sparse
Febrile neutropenia	1.44 (0.82–2.50)	1.43 (0.83–2.49)	1.47 (0.91 to 2.02)	Yes	Scale dependent
Hypogammaglobulinaemia	0.08 (0.04–0.16)	0.08 (0.04–0.16)	0.25 (−0.56 to 1.07)	Yes	Scale dependent
Neutropenia	0.16 (0.10–0.27)	0.16 (0.10–0.27)	−0.76 (−1.43 to −0.10)	Yes	Concordant
Neutrophil count decreased	0.22 (0.07–0.74)	0.25 (0.08–0.78)	−0.87 (−2.50 to 0.76)	No	Scale dependent; sparse
Thrombocytopenia	0.16 (0.08–0.32)	0.17 (0.08–0.33)	−0.99 (−1.94 to −0.05)	Yes	Concordant
Other terms					
Abdominal pain	6.30 (1.60–24.8)	6.82 (1.88–24.7)	−0.31 (−1.94 to 1.32)	No	Scale dependent; sparse
Acute kidney injury	0.41 (0.13–1.37)	0.47 (0.16–1.45)	−0.98 (−2.61 to 0.66)	No	Concordant; sparse
Acute myeloid leukaemia	4.26 (1.03–17.6)	3.91 (1.03–14.8)	2.04 (0.89 to 3.19)	No	Concordant; sparse
Asthenia	1.20 (0.53–2.74)	1.27 (0.57–2.82)	−0.11 (−1.18 to 0.96)	Yes	Concordant
Condition aggravated	45.8 (15.4–136)	43.5 (15.2–124)	0.45 (−0.45 to 1.34)	Yes	Scale dependent
Cough	2.60 (1.21–5.59)	2.69 (1.27–5.68)	0.65 (−0.29 to 1.60)	Yes	Scale dependent
Cytokine release syndrome	0.15 (0.11–0.21)	0.15 (0.11–0.21)	−0.61 (−0.98 to −0.23)	Yes	Concordant
Cytopenia	0.07 (0.02–0.22)	0.08 (0.02–0.23)	−1.18 (−2.81 to 0.45)	No	Scale dependent; sparse

Preferred Term	Ω, primary (95% CI)	Ω, Haldane–Anscombe (95% CI)	Ω_N (95% interval)	≥5 reports per cohort	Classification
Decreased appetite	3.53 (1.57–7.94)	3.62 (1.64–8.00)	0.90 (−0.04 to 1.84)	Yes	Scale dependent
Depressed level of consciousness	33.6 (13.3–84.9)	31.7 (12.8–78.5)	3.64 (3.01 to 4.27)	Yes	Concordant
Diarrhoea	1.04 (0.55–1.94)	1.07 (0.58–1.99)	−0.38 (−1.20 to 0.43)	Yes	Concordant
Disease progression	1.25 (0.57–2.71)	1.29 (0.60–2.75)	0.48 (−0.47 to 1.42)	Yes	Concordant
Disseminated intravascular coagulation	4.21 (0.79–22.4)	4.08 (0.86–19.4)	1.77 (0.14 to 3.40)	No	Scale dependent; sparse
Dizziness	2.85 (0.95–8.57)	3.10 (1.09–8.86)	−0.86 (−2.28 to 0.55)	No	Not robust
Drug ineffective	1.94 (1.20–3.16)	1.98 (1.22–3.20)	−0.07 (−0.69 to 0.54)	Yes	Scale dependent
Dyspnoea	1.88 (0.92–3.87)	1.95 (0.96–3.94)	−0.13 (−1.02 to 0.77)	Yes	Concordant
Fatigue	0.42 (0.19–0.90)	0.44 (0.21–0.94)	−1.29 (−2.36 to −0.22)	Yes	Concordant
Gastrointestinal disorder	5.55 (1.30–23.7)	5.85 (1.50–22.8)	0.40 (−1.23 to 2.04)	No	Scale dependent; sparse
General physical health deterioration	12.3 (6.37–23.9)	12.2 (6.34–23.4)	2.98 (2.39 to 3.57)	Yes	Concordant
Haemoptysis	10.9 (2.28–52.1)	10.3 (2.36–44.4)	2.35 (0.94 to 3.77)	No	Concordant; sparse
Hyperhidrosis	5.81 (1.42–23.8)	6.20 (1.65–23.3)	0.55 (−1.08 to 2.19)	No	Scale dependent; sparse
Hypertension	17.4 (8.23–36.6)	16.9 (8.12–35.2)	2.45 (1.85 to 3.06)	Yes	Concordant
Hypertransaminasaemia	6.27 (1.57–24.9)	6.26 (1.67–23.5)	1.69 (0.28 to 3.11)	No	Concordant; sparse
Hypocalcaemia	2.00 (0.49–8.20)	1.98 (0.52–7.52)	1.21 (−0.20 to 2.63)	No	Concordant; sparse
Hypokalaemia	0.71 (0.23–2.19)	0.77 (0.26–2.25)	0.21 (−1.20 to 1.62)	No	Concordant; sparse
Immune effector cell-associated neurotoxicity syndrome	0.11 (0.05–0.26)	0.11 (0.05–0.26)	−2.27 (−3.34 to −1.20)	Yes	Concordant
Intentional product use issue	0.58 (0.25–1.34)	0.60 (0.26–1.36)	−0.74 (−1.74 to 0.26)	Yes	Concordant
Lethargy	25.6 (7.71–85.3)	24.2 (7.59–77.0)	2.74 (1.74 to 3.74)	Yes	Concordant
Loss of consciousness	7.87 (1.72–36.0)	8.10 (1.96–33.5)	0.64 (−1.00 to 2.27)	No	Scale dependent; sparse
Multiple organ dysfunction syndrome	8.71 (3.71–20.5)	8.35 (3.63–19.2)	3.05 (2.42 to 3.68)	Yes	Concordant
Muscle spasms	3.66 (0.90–14.8)	3.93 (1.06–14.6)	0.03 (−1.61 to 1.66)	No	Not robust
Nausea	2.59 (1.31–5.11)	2.66 (1.36–5.21)	−0.18 (−1.03 to 0.67)	Yes	Scale dependent
Neuropathy peripheral	0.20 (0.10–0.40)	0.21 (0.10–0.41)	−0.85 (−1.75 to 0.04)	Yes	Scale dependent
Off label use	0.42 (0.25–0.72)	0.44 (0.26–0.73)	−1.25 (−1.96 to −0.55)	Yes	Concordant
Plasma cell myeloma	0.01 (0.01–0.02)	0.01 (0.01–0.02)	−1.06 (−1.59 to −0.54)	Yes	Concordant
Plasma cell myeloma recurrent	0.50 (0.13–1.87)	0.45 (0.13–1.56)	1.20 (0.25 to 2.14)	No	Scale dependent; sparse
Plasma cell myeloma refractory	0.03 (0.02–0.07)	0.03 (0.02–0.07)	0.41 (−0.38 to 1.19)	Yes	Scale dependent
Plasmacytoma	0.09 (0.04–0.22)	0.09 (0.04–0.21)	1.91 (1.13 to 2.70)	Yes	Scale dependent
Product dose omission issue	1.12 (0.35–3.66)	1.29 (0.43–3.88)	−1.80 (−3.43 to −0.16)	No	Scale dependent; sparse
Product use in unapproved indication	18.2 (4.81–68.8)	18.5 (5.24–65.4)	−0.68 (−2.09 to 0.74)	No	Scale dependent; sparse
Productive cough	4.99 (1.37–18.2)	5.13 (1.50–17.6)	1.49 (0.08 to 2.90)	No	Concordant; sparse
Pruritus	9.89 (3.33–29.4)	10.3 (3.62–29.4)	−0.65 (−1.92 to 0.61)	Yes	Scale dependent
Pulmonary oedema	16.0 (5.81–44.1)	14.8 (5.58–39.3)	3.49 (2.86 to 4.12)	Yes	Concordant
Pyrexia	1.68 (1.16–2.43)	1.69 (1.17–2.43)	1.16 (0.74 to 1.58)	Yes	Concordant
Rash	1.14 (0.50–2.57)	1.20 (0.54–2.65)	−0.46 (−1.53 to 0.61)	Yes	Concordant
Renal failure	3.57 (1.92–6.65)	3.56 (1.93–6.59)	2.09 (1.47 to 2.71)	Yes	Concordant
Renal impairment	1.12 (0.32–3.96)	1.25 (0.38–4.10)	0.19 (−1.44 to 1.83)	No	Concordant; sparse
Respiratory failure	0.44 (0.13–1.53)	0.50 (0.16–1.59)	−0.43 (−2.06 to 1.21)	No	Concordant; sparse

Preferred Term	Ω, primary (95% CI)	Ω, Haldane–Anscombe (95% CI)	Ω_N (95% interval)	≥5 reports per cohort	Classification
Tachycardia	8.31 (4.20–16.4)	8.23 (4.20–16.1)	2.80 (2.17 to 3.44)	Yes	Concordant
Therapy non-responder	1.94 (0.74–5.06)	2.03 (0.80–5.13)	1.02 (−0.13 to 2.18)	Yes	Concordant
Toxic skin eruption	1.51 (0.39–5.88)	1.52 (0.42–5.54)	1.47 (0.06 to 2.88)	No	Scale dependent; sparse
White blood cell count decreased	0.73 (0.26–2.02)	0.77 (0.29–2.06)	−0.44 (−1.70 to 0.83)	Yes	Concordant

Across the 79 Preferred Terms, 76 classifications were unchanged by the Haldane–Anscombe correction and 47 were concordant with the shrinkage Ω; 46 terms had at least five reports in each exposure cohort. Ω_N, shrinkage Ω of Norén et al. ($\log_2$ scale); $\Omega_{025} > 0$, higher-than-expected and $\Omega_{975} < 0$, lower-than-expected reporting. Concordant, same classification under all methods; sparse, fewer than five reports in at least one exposure cohort; scale dependent, classification differs between the multiplicative and additive models; not robust, classification changed by the continuity correction.

Table S7. Drug-role, myeloma-restricted comparator, partner-agent-exclusion, covariate-adjusted, relapsed-disease comparator, competition, and masking analyses.

Analysis and Preferred Term	Ω (95% CI)
Suspect roles only (327 combination, 2718 teclistamab-only, and 15,222 daratumumab-only reports)	
Pneumonia	1.62 (1.14–2.30)
Respiratory tract infection	1.36 (0.67–2.73)
Influenza	3.17 (1.38–7.26)
Myeloma-restricted background (139,684 reports)	
Pneumonia	3.59 (2.54–5.07)
Respiratory tract infection	2.86 (1.48–5.52)
Influenza	3.89 (1.77–8.57)
Cytokine release syndrome	1.03*
ICANS	0.65*
Neutropenia	0.54*
Thrombocytopenia	0.49*
Excluding reports co-listing an immunomodulatory drug or proteasome inhibitor (137 combination reports)	
Pneumonia	1.53 (0.85–2.74)
Covariate-adjusted, within the myeloma-restricted population (162,041 reports)	
Pneumonia	3.08 (2.16–4.39)
Respiratory tract infection	2.67 (1.35–5.29)
Influenza	2.84 (1.28–6.33)
Relapsed-disease daratumumab comparator (4,278 daratumumab-only reports)	
Pneumonia	1.43 (0.99–2.07)
Influenza	2.49 (1.07–5.75)
Competition: infection-containing reports excluded from all cohorts	
Cytokine release syndrome	0.14 (0.09–0.23)
Neutropenia	0.11 (0.03–0.36)
ICANS	Not estimable (2 combination reports)
Thrombocytopenia	Not estimable (2 combination reports)
Masking: CAR T-cell and other bispecific antibody reports removed from the background	
Cytokine release syndrome	0.03*
ICANS	0.01*

Primary estimates are given in main-text Table 3, and definitions in Supplementary Methods, Section S7. Covariates: age group, sex, year of initial receipt, report type, reporter region, and co-reported immunomodulatory drugs, proteasome inhibitors, and corticosteroids. * Point estimate; 95% CI not reported. CAR, chimeric antigen receptor; ICANS, immune effector cell-associated neurotoxicity syndrome.
